# Adoption of a National Prophylactic Anticoagulation Guideline for Hospitalized Pregnant Women With COVID-19: Retrospective Cohort Study

**DOI:** 10.2196/45586

**Published:** 2023-07-28

**Authors:** Yeon-Mi Hwang, Samantha Piekos, Tanya Sorensen, Leroy Hood, Jennifer Hadlock

**Affiliations:** 1 Institute for Systems Biology Seattle, WA United States; 2 University of Washington Seattle, WA United States; 3 Swedish Medical Center Providence Swedish Seattle, WA United States

**Keywords:** anticoagulants stillbirth, COVID-19, electronic health record, low birth weight, NIH antithrombotic therapy guideline, pregnancy, preterm birth, SARS-CoV-2, small for gestational age, thromboprophylaxis

## Abstract

**Background:**

Both COVID-19 and pregnancy are associated with hypercoagulability. Due to the increased risk for thrombosis, the United States National Institute of Health’s recommendation for prophylactic anticoagulant use for pregnant patients has expanded from patients hospitalized for severe COVID-19 manifestation to all patients hospitalized for the manifestation of COVID-19 (no guideline: before December 26, 2020; first update: December 27, 2022; second update: February 24, 2022-present). However, no study has evaluated this recommendation.

**Objective:**

The objective of this study was to characterize prophylactic anticoagulant use among hospitalized pregnant people with COVID-19 from March 20, 2020, to October 19, 2022.

**Methods:**

This was a retrospective cohort study in large US health care systems across 7 states. The cohort of interest was pregnant patients who were hospitalized with COVID-19, without previous coagulopathy or contraindication to anticoagulants (n=2767). The treatment group consisted of patients prescribed prophylactic dose anticoagulation between 2 days before and 14 days after COVID-19 treatment onset (n=191). The control group was patients with no anticoagulant exposure between 14 days before and 60 days after COVID-19 treatment onset (n=2534). We ascertained the use of prophylactic anticoagulants with attention to the updates in guidelines and emerging SARS-CoV-2 variants. We propensity score matched the treatment and control group 1:1 on the most important features contributing to the prophylactic anticoagulant administration status classification. Outcome measures included coagulopathy, bleeding, COVID-19–related complications, and maternal-fetal health outcomes. Additionally, the inpatient anticoagulant administration rate was validated in a nationwide population from Truveta, a collective of 700 hospitals across the United States.

**Results:**

The overall administration rate of prophylactic anticoagulants was 7% (191/2725). It was lowest after the second guideline update (no guideline: 27/262, 10%; first update: 145/1663, 8.72%; second update: 19/811, 2.3%; *P*<.001) and during the omicron-dominant period (Wild type: 45/549, 8.2%; Alpha: 18/129, 14%; Delta: 81/507, 16%; and Omicron: 47/1551, 3%; *P*<.001). Models developed on retrospective data showed that the variable most associated with the administration of inpatient prophylactic anticoagulant was comorbidities prior to SARS-CoV-2 infection. The patients who were administered prophylactic anticoagulant were also more likely to receive supplementary oxygen (57/191, 30% vs 9/188, 5%; *P*<.001). There was no statistical difference in a new diagnosis of coagulopathy, bleeding, or maternal-fetal health outcomes between those who received treatment and the matched control group.

**Conclusions:**

Most hospitalized pregnant patients with COVID-19 did not receive prophylactic anticoagulants across health care systems as recommended by guidelines. Guideline-recommended treatment was administered more frequently to patients with greater COVID-19 illness severity. Given the low rate of administration and differences between treated and untreated cohorts, efficacy could not be assessed.

## Introduction

Both COVID-19 and pregnancy are associated with thrombosis [1-13]. Severe COVID-19 is accompanied by Virchow’s triad of endothelial injury, stasis, and hypercoagulable state: categories of factors contributing to thrombosis [1-10]. The related virus, SARS-CoV-2, directly invades endothelial cells, leading to endothelial injury, exocytosis, and endotheliitis [1-4,8,14,15]. Hospitalization causes stasis of blood flow due to decreased mobility regardless of the presence of the SARS-CoV-2 virus [16]. COVID-19–induced changes in prothrombotic factors are linked to an increased risk of hypercoagulability [5-7,17-26]. Studies have reported elevated factor VIII, elevated fibrinogen, circulating prothrombotic microparticles, neutrophil extracellular traps, and hyperviscosity among severely ill patients with COVID-19 [5-7,17-20]. Multiple meta-analyses studies report high rates of coagulopathy and thrombosis across multiple tissues and organ sites among patients infected with COVID-19 since early in the pandemic [21-26].

Hypercoagulability is a physiologically adaptive mechanism that prevents bleeding from miscarriage, childbirth, and postpartum hemorrhage. Women in pregnancy or the postpartum period are at a 4- to 5-fold higher risk of thromboembolism than nonpregnant women [11-13]. Normal pregnancy is accompanied by increased concentration of factors VII, VIII, X, and von Willebrand factor and by pronounced increases in fibrinogen [12,27,28]. Due to the combination of increased risk of hypercoagulability, the National Institutes of Health (NIH) COVID-19 Treatment Guideline Panel recommends using a prophylactic dose of anticoagulation for pregnant patients who are hospitalized for COVID-19 unless a contraindication exists, since February 24, 2022 [29].

We hypothesized that we would observe a high prophylactic anticoagulant administration rate after the second update in NIH COVID-19 treatment guidelines. As prophylactic anticoagulant was recommended to all hospitalized pregnant patients with COVID-19, we expected a minimal difference in clinical condition between the treatment and control group, leading to minimized confounding by indication. This would allow us to properly evaluate the impact of prophylactic anticoagulants on inpatient pregnant patients with COVID-19. No study yet has evidence for or against the guideline on recommending prophylactic anticoagulants on inpatient pregnant patients with COVID-19 [29].

Here, we aim to characterize prophylactic anticoagulant use among hospitalized pregnant patients with COVID-19. We assessed the difference in the prophylactic anticoagulant use across the timeline of NIH guideline changes and SARS-CoV-2 variants. We evaluated the association between prophylactic anticoagulant use and risks of coagulopathy, COVID-19, and maternal-fetal health outcomes, after addressing the discrepancy in variables that could influence outcomes.

## Methods

### Study Setting and Population

**Figure 1:**
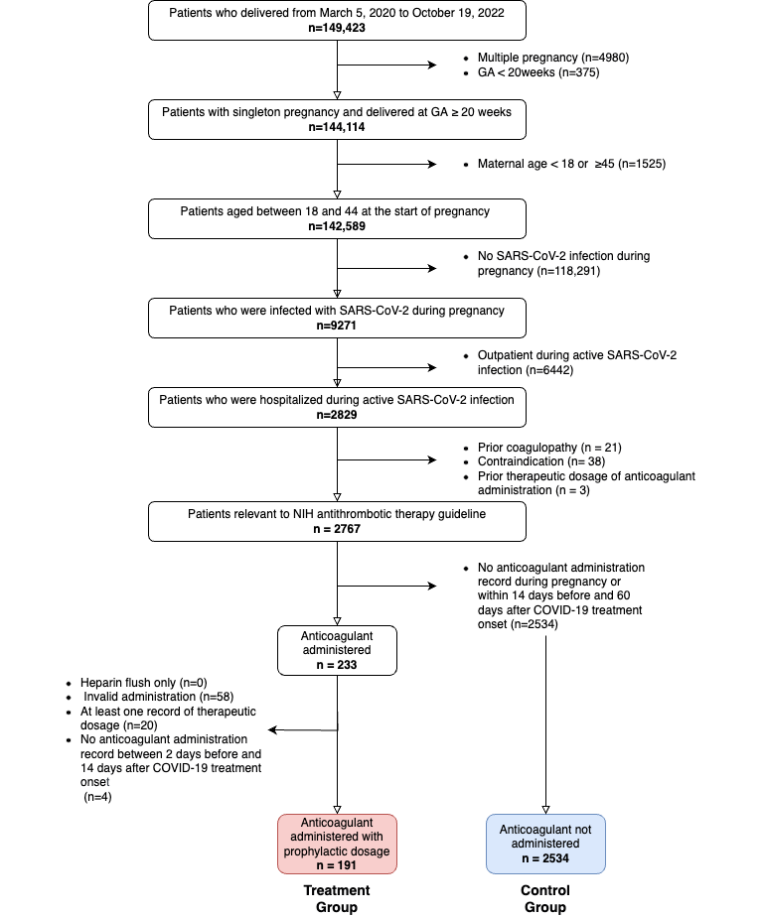
Cohort selection flowchart. We excluded patients with any history of coagulopathy before the time of SARS-CoV-2 infection. We excluded patients with any contradiction to anticoagulant if the diagnosis was between 2 years before the pregnancy and the time of SARS-CoV-2 infection. Contraindications included major bleeding, peptic ulcer, stage 2 hypertension, esophageal varices, intracranial mass, end-stage liver diseases, aneurysm, proliferative retinopathy, and bleeding disorders. We considered administration to be valid unless the administration status was “Canceled Entry,” “Held,” “Missed,” “Automatically Held,” “Held by Provider,” “MAR Hold,” “Stopped,” or “Paused.” GA: gestational age; NIH: National Institutes of Health.

Providence Health and Services and Affiliates (PHSA) is an integrated not-for-profit US community health care system that provides care in urban and rural settings across 7 states: Alaska, California, Montana, Oregon, New Mexico, Texas, and Washington. PHSA service includes 52 hospitals, 1085 clinics, and 120,000 caregivers. We used PHSA electronic health records of pregnant patients who delivered from January 26, 2020, through October 19, 2022 (n=149,423). Multimedia Appendix 1 ([30-36]) provides supplementary methods, tables, and figures. Figure S1 in Multimedia Appendix 1 and Figure 1 describe the cohort selection. Table S1 in Multimedia Appendix 1 defines variables and terminology. We excluded multiple pregnancies and deliveries with a gestational age of less than 20 weeks (n=126,261). We limited our analyses to pregnant patients aged between 18 and 45 years (n=144,114). Our inclusion criterion was COVID-19 diagnosis during the pregnancy period (n=9271). We excluded patients who were not hospitalized with COVID-19 (n=2829). The definition of this exclusion criterion was no overlap of hospitalization stay with an infection period between 7 days before and 14 days after the COVID-19 diagnosis date. This infection period was determined based on previous studies on incubation and symptomatic period [30]. We excluded patients with any coagulopathy event before COVID-19 diagnosis, contraindication to anticoagulant administration, or a record of therapeutic anticoagulant dosage [31] in the past 2 years (Tables S2 and S3 in Multimedia Appendix 1). We defined the remaining patients as our cohort of interest, relevant to the NIH antithrombotic therapy guideline (n=2767). This study was reported following STROBE (Strengthening the Reporting of Observational Studies in Epidemiology) guidelines [37].

### Variables

#### Exposures

Figure S1 in Multimedia Appendix 1 and Figure 1 describe definitions of exposure groups. From the cohort of interest (n=2767), patients with no anticoagulant administration record during pregnancy or within 14 days before and 60 days after the SARS-CoV-2 infection date comprised the control group (no anticoagulant administration group, n=2534). We defined the treatment group (prophylactic anticoagulant administration group, n=191) as patients who received prophylactic anticoagulation between 2 days before and 14 days after the potential COVID-19 treatment onset (Figure S1 and Tables S1, S3, and S4 in Multimedia Appendix 1). Our definition of prophylactic included prophylactic and intermediate dosages (Table S4 in Multimedia Appendix 1) [31].

#### Outcomes

Variables and diagnoses are defined in Tables S1 and S2 in Multimedia Appendix 1. Our primary outcome of interest was coagulopathy. We additionally observed diagnoses relevant to the consequences of COVID-19-associated coagulopathy [8,38,39]. These included thrombosis, pulmonary embolism, thromboembolism, myocardial infarction, skin necrosis or purpura, and stroke (Table S1 in Multimedia Appendix 1). Secondary outcomes were COVID-19 severity and maternal-fetal health outcomes. COVID-19 complications included the need for supplemental oxygen, vasopressor usage, mortality, length of hospital stay, unique diagnosis count, and unique medication count. Maternal-fetal outcomes were stillbirth, preterm birth (PTB), low birth weight (LBW), and small for gestational age. We also assessed the risk of bleeding during pregnancy and postpartum hemorrhage to evaluate the safety of anticoagulant use.

#### Covariates

Maternal characteristics were evaluated, including parity, gravidity, history of preterm delivery, age, race, ethnicity, insurance, pregravid BMI, smoking, illegal drug use, Centers for Disease Control and Prevention Social Vulnerability Index (CDC-SVI), and rural-urban classification (Table S1 in Multimedia Appendix 1). The count of unique diagnoses before COVID-19 was used to reflect the patient’s previous comorbidity. Missing values were imputed using the cohort median. COVID-19–related features assessed were vaccination, previous SARS-CoV-2 infection, presumed SARS-CoV-2 variant, timing of SARS-CoV-2 infection, and stage of NIH COVID-19 antithrombotic therapy guidelines (Supplementary Methods and Table S5 in Multimedia Appendix 1). We defined the stages of guidelines based on major updates in NIH antithrombotic therapy guidelines on pregnant women [29]. Up until December 20, 2020, there was no specific guideline for pregnant women. On December 21, 2020, there was an update to recommend the prophylactic use of anticoagulants among pregnant patients with severe COVID-19. On February 24, 2022, a second update expanded the recommendation to any hospitalized patient with COVID-19.

### Analyses

#### Descriptive Statistics

*P* value was calculated using the chi-square test and the 2-sample *t* test. Multiple testing error was corrected using Bonferroni correction. We performed pairwise Pearson correlation between variables using python library scipy (version 1.6.2). We ranked the count of diagnoses each exposure group received between COVID-19 diagnosis and delivery.

#### Classification Model, Feature Importance, and Propensity Score Matching

We conducted a classification model and feature importance analysis to investigate the differences between the treatment and control groups, and to select variables to be addressed in propensity score matching. Using 28 covariates (Table S1 in Multimedia Appendix 1), we built classification models, including logistic regression, random forest, and gradient boosting machine (GBM) models (Supplementary Methods in Multimedia Appendix 1). We applied random undersampling to address a class imbalance between the control and treatment groups. Due to the small sample size, we leveraged leave-one-out cross-validation. We then used Gini feature importance and SHAP (Shapley additive explanation) [40] to identify which covariates were most important for classifying the administration of prophylactic anticoagulant during a COVID-19–related hospitalization in the best-performing model (Supplementary Methods in Multimedia Appendix 1). We evaluated whether the model trained with these top features achieved comparable performance to the original model with 28 features. We then performed propensity score matching to control for these top features between the treatment and control groups. Compared to other propensity score methods and covariate adjustment methods, propensity score matching provided exceptional covariant balance across most circumstances [41]. Given the extreme weights between treatment and control, the inverse probability treatment weighting method (IPTW) was considered unsuitable for this analysis. K-nearest neighbors (k=1) were used to match with replacement across covariates using propensity logit and caliper of 0.2 using Python library PsmPy (version 0.2.8) to identify patients most similar on the top features and generate a matched control group [42]. The number of neighbors (k) and caliper threshold value were selected based on recommendations from previous studies [43,44]. The effect size of these matched features was evaluated using Cohen *d* score before and after matching with a score of <0.2 indicating a small effect size [45]. We evaluated outcome differences between the treatment and matched control groups using Fisher exact test for categorical variables and the Mann-Whitney *U* test for continuous variables.

#### Sensitivity Analysis

We assessed the influence of illness severity at the time of SARS-CoV-2 infection using the count of medication 3 days before and 3 days after the potential COVID-19 treatment onset. These counts of medications are measures of relative clinical severity in a generally healthy patient population when patients are unlikely to need mechanical ventilation. Although they are imperfect proxies, they have been shown to provide valuable insight, especially in COVID-19–related research. The date range for collecting the medication count was selected based on the distribution of the time gap between the anticoagulant administration date and the COVID-19 treatment onset date (Figure S3 in Multimedia Appendix 1). We conducted propensity score matching on this medication count at the time of SARS-CoV-2 infection, in addition to the best-performing features. We also addressed the influence of maternal age and pregravid BMI, as these variables are known risk factors for coagulopathy.

### Investigation of Inpatient Anticoagulant Administration Rate Across Multiple Health Care Systems Using Truveta

We calculated the rate of the inpatient anticoagulant administration rate in the Truveta patient population (Figure S2 in Multimedia Appendix 1). Truveta is a consortium of 28 health care systems, including PHSA, providing patient care in over 20,000 clinics and 700 hospitals across 43 states [46]. Similar data fields across systems are mapped following the common schema referred to as the Truveta Data Model [47]. Among pregnant patients aged between 18 and 45 years and delivered singleton from January 26, 2020, through October 19, 2022, we identified patients with a COVID-19 diagnosis during pregnancy. We excluded patients who did not have any inpatient encounters during the active COVID-19 infection. We further excluded patients who had any anticoagulant use before pregnancy and defined the remaining patients as our Truveta cohort of interest, relevant to NIH antithrombotic therapy guidelines. From this analytic cohort, we calculated patients who were administered inpatient anticoagulants.

### Ethical Considerations

All procedures were reviewed and approved by the institutional review board at the PHSA through expedited review on November 4, 2020 (study number STUDY2020000196). Consent was waived because the disclosure of protected health information for the study involved no more than minimal risk to the privacy of individuals. Patient data in the Truveta Studio were deidentified.

## Results

Our cohort of interest was composed of 2767 patients (Figure S1 in Multimedia Appendix 1 and Figure 1). A total of 191 and 2534 patients were in the treatment and control groups, respectively. The treatment group was older (mean age 30.9 years; *P*=.02), less likely to be vaccinated (33/191, 17%; *P*<.001), less likely to be infected during the third trimester (139/191, 72.8%; *P*<.001), and more likely to be infected with Delta variant (81/191, 42%; *P*<.001). The treatment group was exposed to enoxaparin and heparin only (Table 1). The date difference between the anticoagulant administration date and potential COVID-19 treatment onset (anticoagulant administration date – COVID-19 treatment onset) ranged from –2 to 14 days. A total of 74.9% (143/191) of the treatment group received anticoagulant prescriptions during the first 3 days of COVID-19 treatment (Figure S3 in Multimedia Appendix 1).

**Figure 2:**
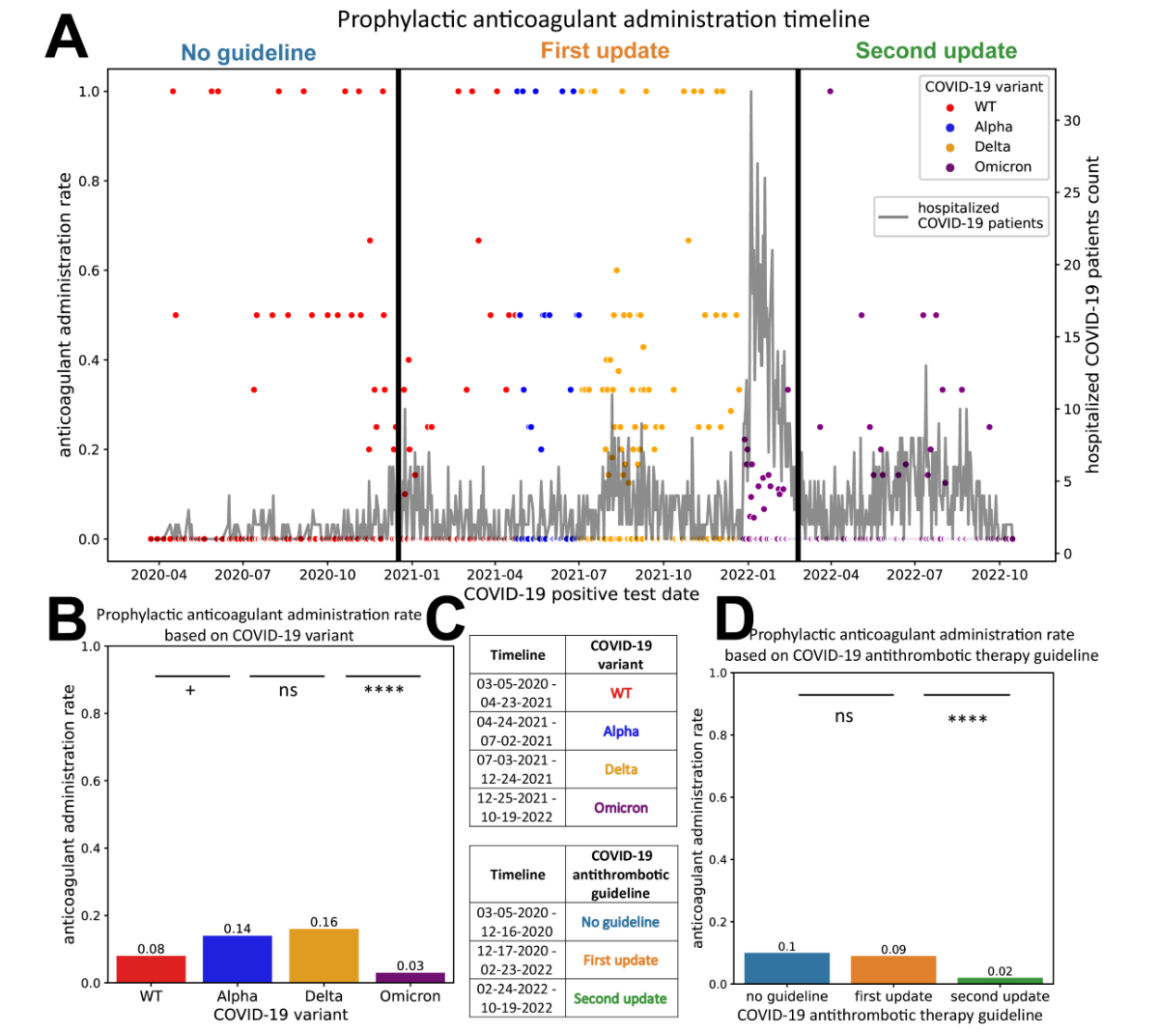
Timeline of prophylactic anticoagulant administration among hospitalized COVID-19 pregnant patients from March 20, 2020, to October 19, 2022. The anticoagulant administration rate is defined as the count of the patients who belong to the treatment group divided by the sum of patients who belong to the treatment group and control group. (A) Timeline of prophylactic anticoagulant administration rate among hospitalized COVID-19 pregnant patients from March 20, 2020, to October 19, 2022. Marked time points are described in part C of the figure. Timeline definitions: red, blue, yellow, and purple dots indicate the prophylactic anticoagulant administration rate among COVID-19 wild type, Alpha, Delta, and Omicron variants, respectively. Grayline chart indicates the count of hospitalized pregnant patients with COVID-19. The first (left) vertical line is on December 17, 2020, the first update date in COVID-19 antithrombotic therapy guidelines to recommend the administration of prophylactic anticoagulants on pregnant patients hospitalized with severe COVID-19 manifestation. The second (right) vertical line is on February 24, 2022, the second update date to expand the recommendation to all pregnant patients hospitalized with COVID-19 manifestation. The overall administration rate of prophylactic anticoagulants was 7% (191/2725) (B) Prophylactic anticoagulant administration rate based on SARS-CoV-2 variant. The SARS-CoV-2 variant was determined based on the period during which each variant was the dominant variant accounting for >50% of cases as part of the Centers for Disease Control and Prevention (CDC) genomic surveillance for SARS-CoV-2 in Region 10 (Alaska, Idaho, Oregon, and Washington; CDC 2022).(C) Prophylactic anticoagulant administration rate was lowest during the omicron-dominant period (Wild type: 45/549, 8.2%; Alpha: 18/129, 14%; Delta: 81/507, 16%; and Omicron: 47/1551, 3%) (C) Timeline definition. Text colors indicate the SARS-CoV-2 variant and COVID-19 antithrombotic therapy guideline time period in parts A, B, and D of the figure. (D) Prophylactic anticoagulant administration rate based on COVID-19 antithrombotic therapy guideline. The first update was to recommend the administration of prophylactic anticoagulants on pregnant patients hospitalized with severe COVID-19 manifestations. The second update expanded the recommendation to all pregnant patients hospitalized with COVID-19 manifestation. Prophylactic anticoagulant administration rate was lowest after the second guideline update (no guideline: 27/262, 10%; first update: 145/1663, 8.7%; second update: 19/811, 2.3%). WT: wild type. ^ns^*P*>.05, *P≤.05, **P≤.01, ***P≤.001, ****P≤.0001.

Figure 2 displays the overall timeline of the prophylactic anticoagulant administration status from March 20, 2020, to October 19, 2022. The overall prevalence rate of prophylactic anticoagulant administration was 7% (191/2725). The administration rate was the lowest when the Omicron variant was dominant (47/1551, 3%) and after the second guideline update (19/811, 2.3%). It was the highest when the Delta variant was dominant (81/507, 16%) and when there was no guideline (27/262, 10%). The top 10 diagnoses of the control group were subcategories of COVID-19 and pregnancy (Figure S4 in Multimedia Appendix 1). The treatment group additionally included COVID-19 complications, such as pneumonia, acute respiratory failure with hypoxia, and hypokalemia (Figure S4 in Multimedia Appendix 1).

Figure S2 in Multimedia Appendix 1 displays the cohort selection procedure in the Truveta patient population. We identified 14,075 patients as our Truveta cohort of interest. Among these patients, 973 (6.9%) patients were administered inpatient anticoagulants.

**Figure 3:**
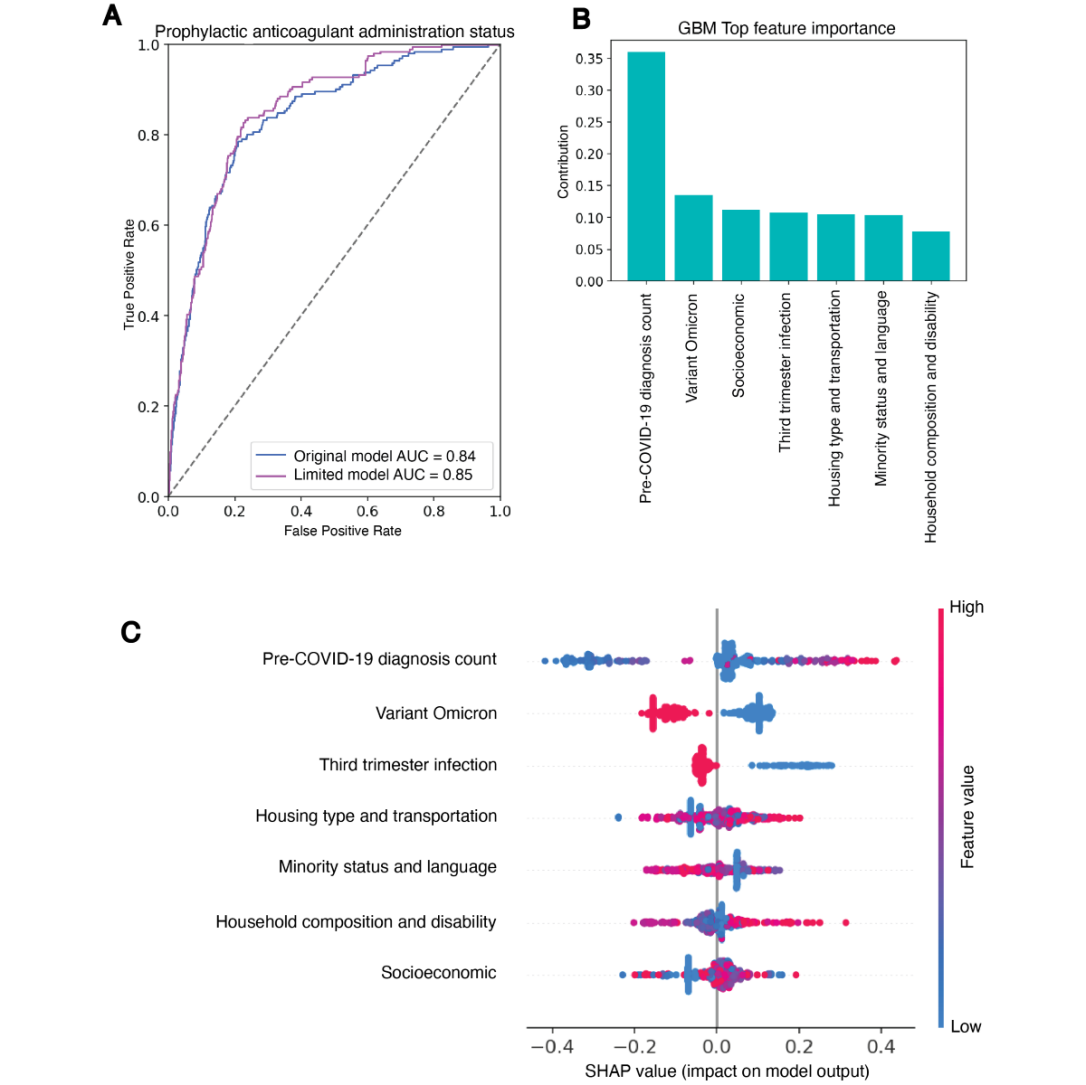
Performance of best-performing classification model and feature importance. (A) Performance of original and limited gradient boosted machine learning model classifying prophylactic anticoagulant administration status among hospitalized pregnant patients with COVID-19. The limited model was trained with the 7 most important features from the original model. The feature importance result of the original model is in Figure S5 in Multimedia Appendix 1. The 95% CI of the original and limited models were (0.81-0.87) and (0.83-0.89), respectively. (B) Feature importance ranking of the limited model. Variables are defined in Table S1 in Multimedia Appendix 1. (C) Shapley permutation explainer of the feature contribution. SHAP value reflects the contribution of the 7 most important features from the gradient boosting models toward classifying prophylactic anticoagulant administration status. SHAP value is the average marginal contribution of a feature value across all permutations of features. Each row represents an individual feature, and the dot represents a sample. The dot color reflects the value of the feature of the sample relative to all samples. The evaluation was done on the sample set composed of a treatment group and a 1:1 randomly undersampled matched control group (n=382). AUC: area under the curve; GBM: gradient boosting machine.

The GBM model had the best performance with an area under the receiver operating characteristics curve (AUC-ROC) of 0.84 (95% CI 0.81-0.87; Figure S5 and Table S6 in Multimedia Appendix 1). Pre–COVID-19 diagnoses count, variant-omicron, socioeconomic status, third-trimester infection, housing type and transportation vulnerability, minority status and language vulnerability, and household composition and disability vulnerability were the most important 7 features (Figures S5 in Multimedia Appendix 1). The GBM model trained with these 7 features reached an AUC-ROC of 0.85 (95% CI 0.83-0.89; Figure S5 in Multimedia Appendix 1 and Figure 3). In the SHAPley figure, red and blue dots indicate high and low feature values. Red dots of pre–COVID-19 diagnoses count were clustered in the positive end of the SHAP axis. Red dots of third-trimester infection and variant Omicron were clustered in the negative SHAP axis. The matched control group (n=188) was generated by propensity score matching on the 7 most important features. These features had small effect sizes after matching (Cohen *d* values <0.2 [45] Table S7 in Multimedia Appendix 1).

The GBM model trained with the 7 top features and initial medication count reached an AUC-ROC of 0.93 (95% 0.91-0.95; Figure S6 in Multimedia Appendix 1). The feature importance showed that initial medication count was the most important feature classifying prophylactic anticoagulant administration status (Figure S6 in Multimedia Appendix 1). The red and blue dots were spread out across extreme negative SHAP values and up to 0.2 SHAP value, but the end of the positive SHAP axis (SHAP value >0.2) was clustered with red dots (Figure S6 in Multimedia Appendix 1).

**Figure 4:**
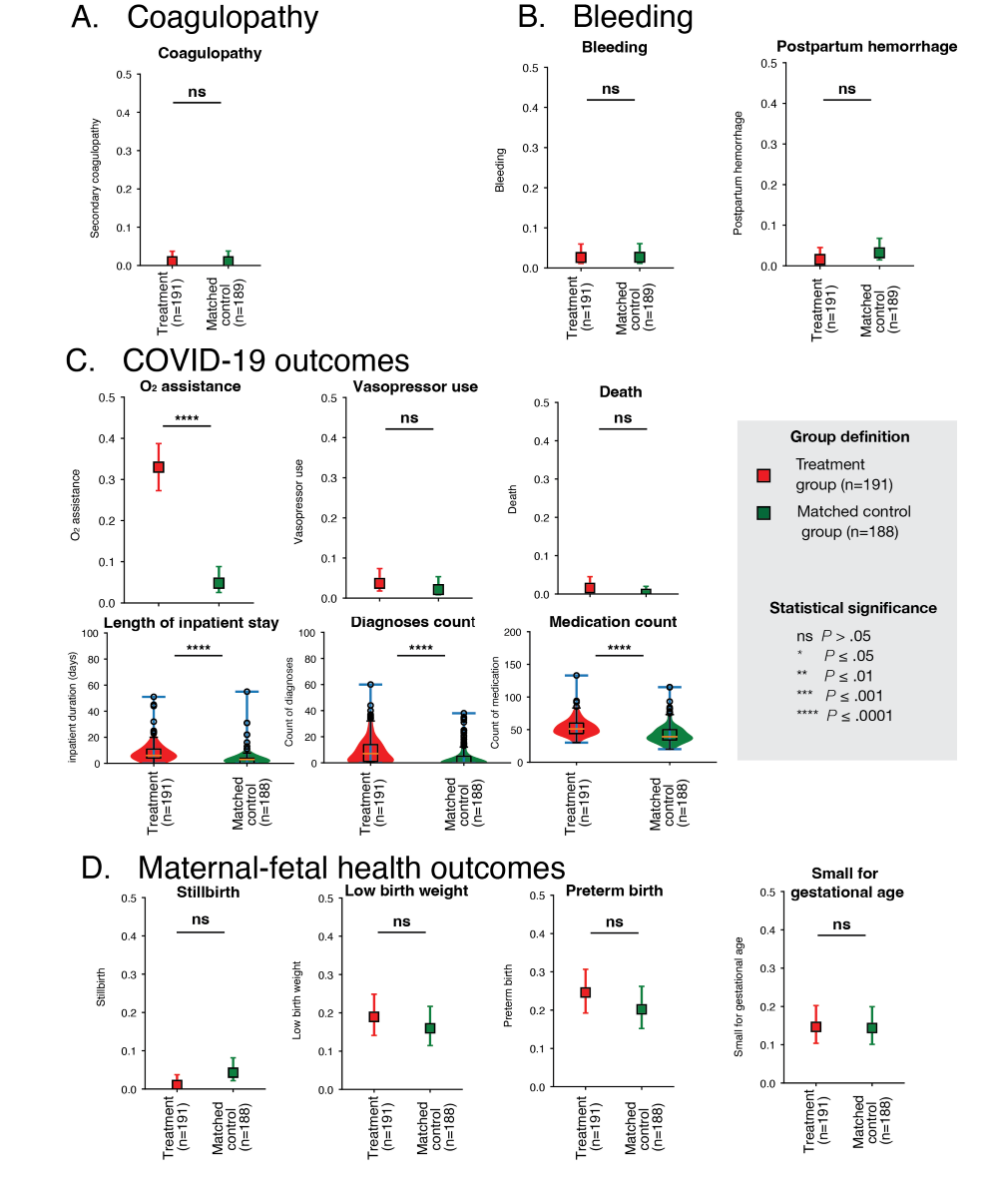
Clinical outcomes of the treatment and matched control. Results are presented in Tables S7 and S8 in Multimedia Appendix 1. Unless specified, the observation cutoff was the delivery date. The observation start point was the anticoagulant exposure date and potential COVID-19 treatment onset date, respectively. (A) Coagulopathy prevalence rate of the treatment and matched control. SNOMED codes we used for coagulopathy diagnosis are listed in Table S1 in Multimedia Appendix 1. (B) Bleeding and postpartum hemorrhage prevalence rate of the treatment and matched control. SNOMED codes we used for bleeding and postpartum hemorrhage diagnoses are listed in Table S1 in Multimedia Appendix 1. Postpartum hemorrhage was collected from the delivery date to +12 weeks postpartum. (C) O_2_ assistance use, vasopressor use, maternal death prevalence rates for the treatment, and matched control groups. Violin plots of the length of inpatient stay (days), medication counts 48 hours after the potential COVID-19 treatment onset, and diagnoses count after the potential COVID-19 treatment onset. The cutoff point was the delivery date. (D) Stillbirth, low birth weight, preterm birth, small for gestational age prevalence rate of the treatment, matched control, and sensitivity analysis matched control group.

Compared with the control group, the treatment group had an increased risk of maternal death (relative risk [RR] not calculable due to the small number of cases; 3/191, 1.6% vs 0/2545, 0%; *P*<.001), O_2_ assistance (RR 9.3, 95% CI 6.9-12.3; 57/191, 33% vs 91/2545, 3.6%; *P*<.001), LBW (RR 2.1, 95% CI 1.5-2.8; 36/190, 19% vs 233/2539, 9.2%; *P*<.001), PTB (RR 2.1, 95% CI 1.5-2.8; 47/191, 25% vs 295/2545, 12%; *P*<.001), and bleeding (RR 2.5, 95% CI 1-6.3; 5/191, 2.6% vs 27/2545, 1.1%; *P*=.08). After matching, risk of LBW (RR 1.2, 95% CI 0.8-1.8; *P*=.50), PTB (RR 1.2, 95% CI 0.8-1.8; *P*=.33), and bleeding (RR 1.0, 95% CI 0.3-3.3; *P*>.99) were reduced and no longer statistically significant. We also did not observe an increase or decrease in risk between the treatment coagulopathy and other maternal-fetal health outcomes (Tables S8 and S9 in Multimedia Appendix 1 and Figure 4). However, the treatment group had significantly worse outcomes regarding COVID-19 illness severity even after the matching. The treatment group had a higher likelihood of supplemental oxygen (RR 6.9, 95% CI 3.5-13.4; 57/191, 30% vs 9/188, 5%; *P*<.001), longer inpatient stay (median 6 vs 3; *P*<.001), higher medication (median 51 vs 39; *P*<.001), and diagnoses count (median 7 vs 0; *P*<.001). The statistical significance remained even in our sensitivity analysis (Tables S8 and S9 in Multimedia Appendix 1) where we additionally addressed the initial illness severity. Maternal age and pregravid BMI did not influence the association between the treatment and outcomes of interest.

**Table 1:** Descriptive statistics of treatment and control group. Variables are defined in Table S1 in Multimedia Appendix 1. This table was generated using tableone PyPI package. *P* value was calculated using the chi-square test and the 2-sample t test. Multiple testing error was corrected using Bonferroni correction.

		Missing, n	Overall (N=2736)	Control group (n=2545)	Treatment group (n=191)	*P* value (adjusted)
Maternal age (years), mean (SD)		0	29.7 (5.7)	29.6 (5.7)	30.9 (5.3)	.02
**Maternal age group (years), n (%)**		0	2736 (100.0)	2545 (100.0)	191 (100.0)	.01
	18-24		647 (23.6)	619 (24.3)	28 (14.7)	
	25-29		758 (27.7)	713 (28.0)	45 (23.6)	
	30-34		797 (29.1)	718 (28.2)	79 (41.4)	
	35-40		435 (15.9)	402 (15.8)	33 (17.3)	
	41-44		99 (3.6)	93 (3.7)	6 (3.1)	
	Unknown		0 (0.0)	0 (0.0)	0 (0.0)	
**Race group, n (%)**		0	2736 (100.0)	2545 (100.0)	191 (100.0)	.02
	American Indian or Alaska Native		45 (1.6)	42 (1.7)	3 (1.6)	
	Asian		166 (6.1)	146 (5.7)	20 (10.5)	
	Black or African American		126 (4.6)	111 (4.4)	15 (7.9)	
	Native Hawaiian or other Pacific Islander		46 (1.7)	38 (1.5)	8 (4.2)	
	White or Caucasian		1466 (53.6)	1381 (54.3)	85 (44.5)	
	Multiracial		150 (5.5)	138 (5.4)	12 (6.3)	
	Other		620 (22.7)	580 (22.8)	40 (20.9)	
	Unknown		117 (4.3)	109 (4.3)	8 (4.2)	
**Ethnic group, n (%)**		0	2736 (100.0)	2545 (100.0)	191 (100.0)	.74
	Hispanic or Latino		1077 (39.4)	1013 (39.8)	64 (33.5)	
	Not Hispanic or Latino		1567 (57.3)	1444 (56.7)	123 (64.4)	
	Unknown		92 (3.4)	88 (3.5)	4 (2.1)	
**Pregravid BMI, n (%)**		1888	848 (100.0)	792 (100.0)	56 (100.0)	.15
	Underweight (below 18.5)		11 (1.3)	11 (1.4)	0 (0.0)	
	Healthy weight (18.5-24.9)		326 (38.4)	314 (39.6)	12 (21.4)	
	Overweight (25-29.9)		241 (28.4)	224 (28.3)	17 (30.4)	
	Obesity (>30)		270 (31.8)	243 (30.7)	27 (48.2)	
**Gravidity, n (%)**		24	2712 (100.0)	2521 (100.0)	191 (100.0)	>.99
	1-5		2529 (93.3)	2354 (93.4)	175 (91.6)	
	>5		13 (6.7)	167 (6.6)	16 (8.4)	
**Parity, n (%)**		24	2712 (100.0)	2521 (100.0)	191 (100.0)	>.99
	0		381 (14.0)	355 (14.1)	26 (13.6)	
	1-5		2298 (84.7)	2136 (84.7)	162 (84.8)	
	>5		33 (1.2)	30 (1.2)	3 (1.6)	
**Preterm history, n (%)**		433	2303 (100.0)	2145 (100.0)	158 (100.0)	>.99
	0		2012 (87.4)	1879 (87.6)	133 (84.2)	
	1		291 (12.6)	266 (12.4)	25 (15.8)	
**Insurance status, n (%)**		1	2736 (100.0)	2545 (100.0)	191 (100.0)	>.99
	Commercial		959 (35.1)	890 (35.0)	69 (36.1)	
	Medicaid		1771 (64.8)	1649 (64.8)	122 (63.9)	
	Medicare		3 (0.1)	3 (0.1)	0 (0.0)	
	Uninsured self-pay		2 (0.1)	2 (0.1)	0 (0.0)	
**Smoking status, n (%)**		0	2736 (100.0)	2545 (100.0)	191 (100.0)	0.49
	No		2488 (90.9)	2322 (91.2)	166 (86.9)	
	Yes		248 (9.1)	223 (8.8)	25 (13.1)	
**Illegal drug use status, n (%)**		0	2736 (100.0)	2545 (100.0)	191 (100.0)	>.99
	No		2399 (87.7)	2226 (87.5)	173 (90.6)	
	Yes		337 (12.3)	319 (12.5)	18 (9.4)	
**Rural-urban classification, n (%)**		538	2198 (100.0)	2029 (100.0)	166 (100.0)	>.99
	Metropolitan		1997 (90.9)	1846 (91.0)	151 (89.3)	
	Micropolitan		129 (5.9)	114 (5.6)	15 (8.9)	
	Rural		31 (1.4)	30 (1.5)	1 (0.6)	
	Small town		41 (1.9)	39 (1.9)	2 (1.2)	
Socioeconomic status vulnerability, mean (SD)		455	0.5 (0.3)	0.5 (0.3)	0.5 (0.3)	>.99
Household composition and disability vulnerability, mean (SD)		452	0.4 (0.3)	0.4 (0.3)	0.5 (0.3)	>.99
Minority status and language vulnerability, mean (SD)		452	0.7 (0.3)	0.7 (0.3)	0.6 (0.2)	.15
Housing type and transportation vulnerability, mean (SD)		455	0.6 (0.3)	0.6 (0.3)	0.6 (0.3)	>.99
**Vaccination status, n (%)**		0	2736 (100.0)	2545 (100.0)	191 (100.0)	<.001
	No		1803 (65.9)	1645 (64.6)	158 (82.7)	
	Yes		933 (34.1)	900 (35.4)	33 (17.3)	
**Previous SARS-CoV-2 infection, n (%)**		0	2736 (100.0)	2545 (100.0)	191 (100.0)	>.99
	No		2690 (98.3)	2500 (98.2)	190 (99.5)	
	Yes		46 (1.7)	45 (1.8)	1 (0.5)	
**Trimester of SARS-CoV-2 infection, n (%)**		0	2736 (100.0)	2545 (100.0)	191 (100.0)	<.001
	First trimester		25 (0.9)	18 (0.7)	7 (3.7)	
	Second trimester		120 (4.4)	75 (2.9)	45 (23.6)	
	Third trimester		2591 (94.7)	2452 (96.3)	139 (72.8)	
**SARS-CoV-2 variant, n (%)**		0	2736 (100.0)	2545 (100.0)	191 (100.0)	<.001
	Wild type		549 (20.1)	504 (19.8)	45 (23.6)	
	Alpha		129 (4.7)	111 (4.4)	18 (9.4)	
	Delta		507 (18.5)	426 (16.7)	81 (42.4)	
	Omicron		1551 (56.7)	1504 (59.1)	47 (24.6)	
**NIH^a^ antithrombotic therapy guideline, n (%)**		0	2736 (100.0)	2545 (100.0)	191 (100.0)	<.001
	No guideline		262 (9.6)	235 (9.2)	27 (14.1)	
	First update		1663 (60.8)	1518 (59.6)	145 (75.9)	
	Second update		811 (29.6)	792 (31.1)	19 (9.9)	
Diagnosis count before SARS-CoV-2 infection, mean (SD)		0	1.9 (3.6)	1.9 (3.7)	2 (3.3)	>.99

^a^NIH: National Institutes of Health.

## Discussion

### Principal Results

To our knowledge, this was the first study evaluating the NIH antithrombotic therapy guideline on pregnant women. We initially hypothesized to observe a high prophylactic anticoagulant administration rate among hospitalized COVID-19 pregnant patients and minimal difference in patient characteristics between the treatment and control groups. However, we identified most patients relevant to NIH antithrombotic therapy guidelines, pregnant women hospitalized with COVID-19, did not receive prophylactic anticoagulant dosage. Instead, patients with higher comorbidity levels, infected in the first or second trimester, and infected with a non-Omicron variant were more likely to receive prophylactic anticoagulant. Despite having more severe COVID-19, the treatment group did not have an elevated risk of coagulopathy, bleeding, and maternal-fetal health outcomes.

### Comparisons With Previous Works, Interpretation, and Implication

The most important and interesting finding was an unexpectedly low prophylactic anticoagulant administration rate among hospitalized COVID-19 pregnant patients across health care systems despite the guideline. A possible explanation for this observation is that clinicians administered prophylactic anticoagulants based on the patient’s COVID-19 illness severity. The initial medication count, a proxy variable for COVID-19 illness severity at COVID-19 treatment onset, was the most important feature in the sensitivity analysis model classifying prophylactic anticoagulant administration status. It also enhanced the area under the curve score by 0.09 reaching 0.94. Besides, we noticed the treatment group was enriched with diagnoses indicative of severe COVID-19 symptoms, such as pneumonia, acute respiratory failure with hypoxia, and hypokalemia. In contrast, the control group was not. We presume clinicians did not follow the guideline because it was not strongly recommended and lacked studies to support it. The rating scheme of the NIH guideline is BIII. B and III, respectively, indicate a moderate recommendation and expert opinion evidence. This means no study yet has evidence for or against this recommendation [42]. Few studies describing COVID-19-positive pregnant patients have reported prophylactic use of anticoagulants during hospitalization. In these studies, the sample size was small, ranging from 9 to 20, and complications following the treatment were not evaluated [48-50].

We noted an elevated risk of respiratory assistance among the treatment group. This was inconsistent with previous studies. Although there was no study on pregnant patients, there have been multiple observational studies supporting prophylactic anticoagulant use on nonpregnant hospitalized patients with COVID-19. Studies showed that prophylactic anticoagulants improved freedom from intubation and lowered mortality, indicating better COVID-19 outcomes [51-53]. In these studies with a nonpregnant population, including men and an older population, the rate of prophylactic anticoagulant administration ranged from 45% (1959/4389) to 84% (3627/4297), whereas that of our study was 7% (191/2736). In addition, in our study, prophylactic anticoagulant administration was highly correlated with the comorbidity level before the SARS-CoV-2 infection and the initial illness severity. We do not think the increased risk in respiratory assistance in our study was influenced by the exposure to anticoagulants, but rather a byproduct of failure to overcome the confounding by indication.

Although we could only partially address the confounding by indication, we did not observe elevated risks of coagulopathy, bleeding, and maternal-fetal health outcomes among the treatment group. Given that severe COVID-19 is associated with coagulopathy and adverse pregnancy outcomes [1-10,54,55], this finding was somewhat promising as we expected worse outcomes in coagulopathy and maternal-fetal health outcomes. Based on our findings, we cannot support the benefit of the guideline, but can, at least, support its potential safety regarding maternal-fetal health outcomes. The treatment group had a similar likelihood to deliver low birthweight, preterm, and small for gestational age babies. This observation was less limited with a small sample size, compared to rare outcomes of interest, due to the high incidence rate of cases across groups. Considering this is the first study to evaluate the guideline, our study can contribute to our knowledge in treating hospitalized pregnant patients with COVID-19. Future research should be conducted in larger and various study settings and minimize the confounding by indication to understand better the risk, safety, and benefit of the treatment.

### Strengths and Limitations

The small percentage of patients receiving guideline-recommended treatment was both an interesting observation and a major limitation. We expected to observe a high prophylactic anticoagulant administration rate after the second guideline update as all hospitalized COVID-19 pregnant patients were relevant, but only 7% (191/2736) were administered prophylactic anticoagulants. This led to confounding by indication. The treatment group had higher pre-COVID-19 comorbidity levels and included more critically ill patients. Although we attempted to minimize the bias using propensity score matching, we could not overcome it due to the unexpectedly small sample size and lack of appropriate variables reflecting COVID-19 illness severity at the time of infection. This study was conducted on retrospective structured data, which is insufficient to determine medical reasoning. In addition, individuals had a wide range of anticoagulant administration start times before and after the potential COVID-19 treatment onset. As covariates that occurred after the exposure are inappropriate for the propensity score method, we excluded them from the main matching model. Nevertheless, we performed sensitivity analysis on the impact of the initial medication count to partially address the COVID-19 illness severity at the time of treatment onset. Last, we did not verify whether the heightened risk of O_2_ assistance among the treatment group resulted from confounding by indication or not. We assumed it was due to confounding by indication based on the previous studies [51-53] on hospitalized patients with COVID-19. More generally, electronic health record data are known to contain errors; inpatient medication administration records are carefully tracked and unlikely to be omitted, but it is not uncommon for comorbidities to be undiagnosed, unreported, or listed in more than one way. To address all of these limitations, a similar study should be conducted in a larger prospective cohort, with appropriate variables to assess COVID-19 illness severity at the time of admission.

This was the first study assessing the prophylactic use of anticoagulants on hospitalized patients after the guideline update on February 24, 2022. Although the guideline recommends on all hospitalized patients regardless of COVID-19 severity, no study to date has assessed the exposure of anticoagulants among COVID-19 hospitalized patients. Another strength of our study includes adjustments for social and economic risk factors. We adjusted for differences between CDC-SVI scores and rural and urban classifications in the treatment and control groups based on the census tract level. Social vulnerability and rural health disparities are significant risk factors for adverse pregnancy outcomes and severe COVID-19 symptoms [56-63]. As the census tract is more granular than the county level, it captures the environmental factors to which patients have been exposed more accurately. Indeed, all 4 CDC-SVI indexes were important predictors of anticoagulant administration status. This study was conducted on a COVID-19 maternity population that has been investigated by other researchers [64,65], allowing deeper insight into the study setting and population.

We validated the number of inpatient anticoagulant administration rates in Truveta’s patient population, comprised of various health care systems. Overall inpatient anticoagulant administration rates in Truveta were similar to those observed in PHSA. This indicates that the unexpectedly low adaptation rate of NIH recommendations was not limited to the PHSA health care system. Here, we could not completely replicate our analysis as Truveta and Providence data models were different, and the leading researcher of this study had limited access to Truveta Studio. We did not exclude patients who were contraindicated to anticoagulants and did not differentiate between prophylactic and therapeutic dosage. Nevertheless, we expect our final estimation of the inpatient anticoagulant administration rate on the Truveta patient population would not drastically change based on the number we observed from the cohort selection procedure in the PHSA population.

### Conclusions

We found that in a population of patients from multiple health care systems nationwide, only a small percentage of hospitalized pregnant patients received the prophylactic anticoagulation recommended by the NIH antithrombotic therapy guideline. Guideline recommended prophylactic anticoagulants were administered more frequently to patients with greater COVID-19 illness severity. We were not able to show the efficacy of prophylactic anticoagulation. However, anticoagulation was not associated with elevated risks of coagulopathy, bleeding, and maternal-fetal health outcomes despite the worse health conditions of the treatment group. This study cannot make a recommendation to administer prophylactic anticoagulant to hospitalized COVID-19 pregnant patients. However, as this was the first study characterizing prophylactic anticoagulant use after the NIH guideline update on February 24, 2022, these results can help inform patient-specific clinical decisions. An observational study with a larger sample size should be considered to further evaluate outcomes with prophylactic anticoagulation.

## Appendix

Multimedia Appendix 1 Supplementary methods, tables, and figures.

## Abbreviations

AUC-ROC: area under the receiver operating characteristics curve
CDC-SVI: Centers for Disease Control and Prevention Social Vulnerability Index
GBM: gradient boosting machine
LBW: low birth weight
NIH: National Institutes of Health
PHSA: Providence Health and Services and affiliates
PTB: preterm birth
RR: relative risk
SHAP: Shapley additive explanation
STROBE: Strengthening the Reporting of Observational Studies in Epidemiology

## Data Availability

Results have been aggregated and reported within this paper to the extent possible while maintaining privacy from personal health information as required by law. Providence Health and Services and Affiliates data are archived within Providence Health and Services and Affiliates in a Health Insurance Portability and Accountability Act (HIPAA)–secure audited computer environment. Truveta data are available to Truveta subscribers. Requests to access these data sets can be directed to Truveta Inc., info@truveta.com. All clinical logic for data extraction has been shared within the paper and Multimedia Appendix 1. The code is publicly available on GitHub [66].
