# Supplementary material for: Adoption of a National Prophylactic Anticoagulation Guideline for Hospitalized Pregnant Women With COVID-19: Retrospective Cohort Study
Source: JMIR Public Health Surveill. 2023 Jul 28;9:e45586. doi: 10.2196/45586 (PMC10389076; doi:10.2196/45586)
Supplement: Multimedia Appendix 1 [file publichealth_v9i1e45586_app1.docx]

Supplementary Materials

*Adoption of a National Prophylactic Anticoagulation Guideline for Hospitalized Pregnant Women with COVID-19: Retrospective Cohort Study*

[Supplementary Methods 2](#_Toc141081227)

[CDC Social Vulnerability Index (CDC-SVI) and rural-urban classification 2](#_Toc141081228)

[Classification models 2](#_Toc141081229)

[Supplementary Tables 2](#_Toc141081230)

[Table S1 Variable definitions 2](#_Toc141081231)

[Table S2 Diagnosis SNOMED code 5](#_Toc141081232)

[Table S3 Medication ingredient RxNorm codes 5](#_Toc141081233)

[Table S4 Prophylactic, intermediate, and therapeutic dosage of anticoagulant 6](#_Toc141081234)

[Table S5 Timeline of dominant SARS-CoV-2 variant in the U.S Western states used in this study 6](#_Toc141081235)

[Table S6 Propensity score matching effect size comparison for matched and sensitivity analysis matched control groups 6](#_Toc141081236)

[Table S7 Propensity score matching effect size comparison for matched control and sensitivity analysis matched control groups 7](#_Toc141081237)

[Table S8 Outcome statistics for treatment, control, matched control, and sensitivity analysis matched control group 7](#_Toc141081238)

[Table S9 Outcome comparison between treatment group and control, matched control, and sensitivity analysis matched control group 10](#_Toc141081239)

[Table S10 Influence of maternal age and pregravid BMI on relations between prophylactic anticoagulant administration and risks of outcomes 12](#_Toc141081240)

[Supplementary Figures 13](#_Toc141081241)

[Figure S1 Timeline and exposure group definition 13](#_Toc141081242)

[Figure S2 Truveta cohort selection flow chart 14](#_Toc141081243)

[Figure S3 Distribution of the time gap (in days) between COVID-19 treatment onset and anticoagulant administration 15](#_Toc141081244)

[Figure S4 Top 10 diagnoses of treatment and control group during the period between SARS-CoV-2 infection and delivery date 16](#_Toc141081245)

[Figure S5 Classification model performance 18](#_Toc141081246)

[Figure S6 Sensitivity analysis result of classification model performance, feature importance, and propensity score matching result 19](#_Toc141081247)

[Figure S7 Pairwise Pearson correlation plot between variables 21](#_Toc141081248)

[Figure S8. Pearson correlation between individual variables and prophylactic anticoagulant administration status 22](#_Toc141081249)

# Supplementary Methods

## CDC Social Vulnerability Index (CDC-SVI) and rural-urban classification

Patient’s address was converted to a U.S. census tract. We imported publicly available CDC-SVI[[32]](https://paperpile.com/c/KBCGQG/MxE6j) and rural-urban classification[[33]](https://paperpile.com/c/KBCGQG/oXwc9) resources from the CDC Agency for Toxic Substance and Disease Registry and the U.S. Department of Agriculture Economic Research Service into our HIPPA-compliant workspace. Individuals’ census tract was mapped to CDC SVI and rural-urban classification code. CDC-SVI represents the percentile ranking of each census tract on 15 social factors. Social factor themes include socioeconomic status, household composition, race/ethnicity/language, and housing/transportation. CDC scores range from 0 to 1. A higher SVI score indicates a higher vulnerability of an individual’s U.S. census tract exposed for each theme. For rural-urban classification, we specifically used SecondaryRUCACode2010 (last updated in 2019) to categorize an individual’s census tract into metropolitan (<4), micropolitan (4-6), small town(7-9), and rural (10-99).

## Classification models

Models were generated using python package sklearn (version 1.0.2) with default settings for logistic regression, gradient boosting regression, and random forest. Due to the imbalance of dataset, we randomly undersampled control group to 1:1 match the treatment group. We leveraged leave-one-out-cross-validation (LOOCV). LOOCV is an extreme version of k-fold cross-validation where k equals n. We left one sample as a test set at a time, and this step was repeated for all samples. Model performance was evaluated using mean absolute error, mean squared error, root mean squared error, area under the curve of receiver operating characteristics (AUC-ROC), AUC of the precision-recall curve, R^2^. Gini feature importance was used to assess the marginal contribution and contribution of an individual feature to the model. Due to the computational cost, we did not use our final model to run Shapley additive explanation (SHAP). We 1:1 randomly undersampled the control group to match the treatment group. We leveraged SHAP on these 1:1 matched samples to evaluate the average marginal contribution of a feature value across all permutations of features. This provided insight into the degree of influence of the feature on an individual's classification prophylactic anticoagulant administration status for the gradient boosting model, the final model.

# Supplementary Tables

## Table S1 Variable definitions

| **Category** | **Variable** | **Definitions** |
| --- | --- | --- |
| **COVID-19 related** | SARS-CoV-2 infection date | Either the date of COVID-19 diagnosis or positive SARS-CoV-2 PCR/NAAT test, whichever preceded |
|  | COVID-19 treatment onset | Starting time of COVID-19 treatment. If patient was hospitalized more than 7 days before the SARS-CoV-2 infection date, SARS-CoV-2 infection date was defined as COVID-19 treatment onset. If patient was hospitalized less than 7 days before or after the COVID-19 infection date, patient's admission date was defined as COVID-19 treatment onset. |
|  | Active SARS-CoV-2 infection period | -2~14 days from the SARS-CoV-2 infection date |
|  | Hospitalization with COVID-19 | Any overlap of hospitalization and active SARS-CoV-2 infection period |
|  | SARS-CoV-2 variant | Dominant variant at the time of SARS-CoV-2 infection. Variant was considered predominant when the variant account for >50% cases as part of the CDC genomic surveillance for SARS-CoV-2 in region 10 (Alaska, Idaho, Oregon, and Washington); Wild Type, Alpha, Delta, Omicron |
|  | Trimester of SARS-CoV-2 infection | Trimester of SARS-CoV-2 infection date; first, second, third trimester |
|  | NIH antithrombotic therapy guideline | Status of NIH antithrombotic therapy guideline on pregnant women. There was no specific guideline on pregnant women until December 16, 2020. The first major update was on December 17, 2020, recommending prophylactic anticoagulant use on pregnant women hospitalized with severe COVID-19 manifestation. The second major update was on February 24, 2022 recommending prophylactic anticoagulant use on pregnant women hospitalized with COVID-19 manifestation; no guideline, first update, second update |
|  | Vaccination status | History of COVID-19 vaccination (Pfizer-BioNTech BNT162B2, Moderna mRNA-1273, Janssen Ad26.COV2.S) based on immunization record; 0,1 |
|  | Prior infection status | History of COVID-19 based on diagnosis or positive SARS-CoV-2 PCR/NAAT test; 0,1 |
| **COVID-19 illness severity** | Oxygen assistance | Any oxygen assistance after SARS-CoV-2 infection and before delivery; 0, 1 (low flow, high flow oxygen devices) |
|  | Vasopressor use | Vasopressor administration record after SARS-CoV-2 infection and before delivery; 0, 1 |
|  | Death | Maternal death occurred after SARS-CoV-2 infection and before delivery |
|  | Days in hospital after COVID-19 diagnosis | Length of hospitalization stay from SARS-CoV-2 infection to delivery date |
|  | Unique medication count | Count of unique medication ingredient from SARS-CoV-2 infection to delivery date |
|  | Unique diagnosis count | Count of unique diagnosis from SARS-CoV-2 infection to delivery date |
| **Demographic** | Race | Race noted in medical record. Missing values were encoded as Unknown; American Indian or Alaska Native, Asian, Black or African American, Native Hawaiian or Other Pacific Islander, White or Caucasian, Multiracial, Other, Unknown |
|  | Ethnicity | Ethnicity noted in medical record. Missing values were encoded as unknown; Hispanic or Latino, Not Hispanic or Latino, Unknown |
|  | Maternal age | Maternal age at the start of pregnancy; 18 ~ 24, 25 ~ 29, 30 ~ 34, 35 ~ 40, 41 ~ 44 |
|  | Pregravid BMI | Pregravid Body Mass Index (kg/m2). Missing value was encoded as unknown; Underweight (<18.5 BMI), Normal (18.5 - 24.9 BMI), Overweight ( 25.0 - 29.9 BMI), Obese (>30.0) |
|  | Insurance status | Commercial, Medicaid, Medicare, Uninsured-Self-Pay |
|  | Smoker | Self-reported smoking status; 0,1 |
|  | Illegal drug use | Self-reported illegal drug use status; 0,1 |
|  | Preterm history | History of preterm delivery; 0,1 |
|  | Parity | Number of times a patient has delivered a fetus older than 20 weeks of gestation prior to the current pregnancy; 0, 1~5, 6 |
|  | Gravidity | Number of times a patient has been pregnant; 0, 1~5, 6 |
| **Comorbidity** | Diagnosis count before COVID-19 | Number of unique diagnoses from two year before the start of the pregnancy to SARS-CoV-2 infection date |
|  | Initial medication count | Number of unique medications during -3~+3 days from the COVID-19 treatment onset |
| **Geographical features** | Socioeconomic status | CDC SVI Socioeconomic (RPL_THEME1) theme ranking mapped to patient's U.S. Census tract; scores are 0-1. score of 0 and 1 indicate low and high level of social vulnerability regarding socioeconomic status |
|  | Housing composition | CDC SVI Housing Composition & Disability (RPL_THEME2) theme ranking mapped to patient's U.S. Census tract; scores are 0-1. score of 0 and 1 indicate low and high levels of social vulnerability regarding housing composition. |
|  | Minority status and language | CDC SVI Minority Status & Language (RPL_THEME3) theme ranking mapped to patient's U.S. Census tract; scores are 0-1. score of 0 and 1 indicate low and high levels of social vulnerability in terms of minority status and language. Census tract with high RPL_THEME3 score is enriched with residents of minority (non-White) and/or have low English language skills |
|  | Housing type and transportation | CDC SVI Housing Type & Transportation (RPL_THEME4) theme ranking mapped to patient's U.S. Census tract; scores are 0-1. score of 0 and 1 indicate low and high levels of social vulnerability regarding housing type and transportation. Census tract with high RPL_THEME4 score is area of lower income housing and/or population dense housing options |
|  | Rural/urban categorization | U.S. Department of Agriculture Economic Research Service (USDA ERS) Rural-Urban Commuting Area (RUCA) codes; SecondaryRUCACode2010 (last updated in 2019) were mapped using patient U.S. Census Tract; Categorized as Metropolitan (> 4 score), Micropolitan (4 - 6 score), Small Town (7 - 9 score), Rural (10 - 99 score), or Unknown |
| **Maternal-fetal health outcomes** | Low birth weight | Infant birth weight ≤ 2,500g |
|  | Preterm birth | Infant gestational age (GA) at birth < 37 weeks |
|  | Small for gestational age | Infant birth weight < 10th percentiles for infants of same GA |
|  | Stillbirth | Fetal demise in the womb ≥ GA 20 weeks |

CDC: Center for Disease Control and Prevention

SVI: Social Vulnerability Index

## Table S2 Diagnosis SNOMED code

| **Category** | **Diagnosis** | **SNOMED hypernym code** |
| --- | --- | --- |
| **Coagulopathy** | Coagulopathy | 64779008 |
|  | Thrombosis | 439127006 |
|  | Arterial thrombosis | 65198009 |
|  | Venous thrombosis | 111293003 |
|  | Deep vein thrombosis | 128053003 |
|  | Thromboembolism | 371039008 |
|  | Pulmonary embolism | 59282003 |
|  | Pulmonary necrosis | 7159003 |
|  | Skin necrosis/purpura | 95347000, 423902002 |
|  | Stroke | 230690007 |
|  | Myocardial infarction | 22298006 |
| **Bleeding** | Bleeding | 131148009 |
|  | Postpartum hemorrhage | 47821001 |
| **Anticoagulant contraindications** | Peptic ulcer | 1320003 |
|  | Stage 2 hypertension | 827068008 |
|  | Esophageal varices | 26870008 |
|  | Intracranial mass | 85974009 |
|  | End stage liver disease | 7082480004 |
|  | Aneurysm | 85659009 |
|  | Proliferative retinopathy | 430801000124103 |
|  | Major bleeding | 74474003 (gastrointestinal)  98478000 (intraocular)  1386000 (intracranial)  95549001 (retroperinatal) |

All descendant SNOMED codes of listed codes were included.

## Table S3 Medication ingredient RxNorm codes

| **Drug** | **RxNorm Code** | **RxNorm Ingredient** |
| --- | --- | --- |
| Anticoagulant(heparin) | 1009 | Antithrombin III |
|  | 280611 | Bemiparin |
|  | 67109 | Dalteparin |
|  | 78484 | Danaparoid |
|  | 67108 | Enoxaparin |
|  | 5224 | Heparin |
|  | 67031 | Nadroparin |
|  | 69528 | Parnaparin |
|  | 75960 | Reviparin |
|  | 69646 | Tinzaparin |
| Anticoagulant(other) | 154 | Acenocoumarol |
|  | 1364430 | Apixaban |
|  | 15202 | Argatroban |
|  | 1927851 | Betrixaban |
|  | 60819 | Bivalirudin |
|  | 1037042 | Dabigatran etexilate |
|  | 114934 | Desirudin |
|  | 1598 | Dicumarol |
|  | 1599538 | Edoxaban |
|  | 50097 | Fluindione |
|  | 321208 | Fondaparinux |
|  | 237057 | Lepirudin |
|  | 8150 | Phenprocoumon |
|  | 8130 | Phenindione |
|  | 1114195 | Rivaroxaban |
|  | 163426 | Tioclomarol |
|  | 11289 | Warfarin |
| Vasopressor | 3616 | Dobutamine |
|  | 3628 | Dopamine |
|  | 3966 | Ephedrine |
|  | 3992 | Epinephrine |
|  | 6963 | Midodrine |
|  | 7512 | Norepinephrine |
|  | 8163 | Phenylephrine |
|  | 11149 | Vasopressin (USP) |

## Table S4 Prophylactic, intermediate, and therapeutic dosage of anticoagulant

| **Heparin** | **Dose level** | **Dose** |
| --- | --- | --- |
| LMW heparin | Prophylactic | Enoxaparin 40mg SC once daily |
|  | Intermediate | Enoxaparin 40mg SC once daily, increases as pregnancy progresses to 1mg/kg once daily |
|  | Therapeutic | Enoxaparin 1mg/kg SC every 12 hours |
| Unfractionated heparin | Prophylactic | 5000 unit SC every 12 hours |
|  | Intermediate | First trimester: 5000-7500 unit SC every 12 hours |
|  |  | Second trimester: 7500-10000 unit SC every 12 hours |
|  |  | Third trimester: 10000 unit SC every 12 hours |
|  | Therapeutic | Continuous IV infusion or SC dose every 12 hours |

Anticoagulation dose during pregnancy[[31]](https://paperpile.com/c/KBCGQG/JIM7q)

## Table S5 Timeline of dominant SARS-CoV-2 variant in the U.S Western states used in this study

| **Dominant Variant** | **Start Date** | **End Date** |
| --- | --- | --- |
| Wild-Type | 3/5/2020 | 4/23/2021 |
| Alpha | 4/24/2021 | 7/2/2021 |
| Delta | 7/3/2021 | 12/24/2021 |
| Omicron | 12/25/2021 | 10/19/2022 |

Variant was considered dominant when a specific variant exceeded 50% of cases of U.S. Region 10 (Alaska, Idaho, Oregon, Washington) CDC's national genomic surveillance system[[34]](https://paperpile.com/c/KBCGQG/hpUxn)

## Table S6 Propensity score matching effect size comparison for matched and sensitivity analysis matched control groups

|  | **Mean Absolute Error** | **Mean Squared Error** | **Root Mean Squared Error** | **ROC-AUC** | **Coefficient of determination** | **Precision-recall AUC** |
| --- | --- | --- | --- | --- | --- | --- |
| **Logistic Regression** | 0.26 | 0.26 | 0.51 | 0.72 | -2.98 | 0.30 |
| **Random Forest** | 0.36 | 0.18 | 0.43 | 0.80 | -1.80 | 0.24 |
| **Gradient Boost Machine** | 0.33 | 0.17 | 0.41 | 0.84 | -1.54 | 0.31 |
| **Gradient Boost Machine Limited** | 0.31 | 0.16 | 0.40 | 0.85 | -1.47 | 0.31 |

ROC Receiver operating characteristics, AUC Area under the curve

## Table S7 Propensity score matching effect size comparison for matched control and sensitivity analysis matched control groups

| **Main matching model effect size comparison** | | |
| --- | --- | --- |
| **Variable** | **Before matching effect size** | **After matching effect size** |
| Variant omicron | -0.71 | 0.04 |
| CDC SVI socioeconomic status | -0.10 | -0.10 |
| CDC SVI household composition and disability | 0.08 | -0.14 |
| CDC SVI minority status and language | -0.22 | -0.09 |
| CDC SVI housing type and transportation | -0.07 | -0.11 |
| 3rd trimester infection | -1.09 | -0.04 |
| Pre-COVID-19 diagnoses count | 0.70 | 0.11 |
| **Sensitivity analysis matching model effect size comparison** | | |
| **Variable** | **Before matching effect size** | **After matching effect size** |
| Variant omicron | -0.71 | 0.15 |
| CDC SVI socioeconomic status | -0.10 | -0.12 |
| CDC SVI household composition and disability | 0.08 | -0.25 |
| CDC SVI minority status and language | -0.22 | 0.00 |
| CDC SVI housing type and transportation | -0.07 | -0.14 |
| 3rd trimester infection | -1.09 | -0.01 |
| Pre-COVID-19 diagnoses count | 0.70 | 0.25 |
| COVID-19 initial medication count | -0.06 | 0.14 |

Effect size was calculated using Cohen D’s value. The absolute value of effect size < 0.2 is considered a small effect size.

## Table S8 Outcome statistics for treatment, control, matched control, and sensitivity analysis matched control group

| **Variable** | **Categories** | **Treatment (n=191)** | **Control (n=2545)** | **Matched Control (n=188)** | **SA Matched Control (n=189)** |
| --- | --- | --- | --- | --- | --- |
|  |  |  |  |  |  |
| Secondary Coagulopathy,  n (%) |  | 191 (100.0) | 2545 (100.0) | 188 (100.0) | 189 (100.0) |
|  | No | 189 (99.0) | 2536 (99.6) | 186 (98.9) | 188 (99.5) |
|  | Yes | 2 (1.0) | 9 (0.4) | 2 (1.1) | 1 (0.5) |
| Skin Necrosis/Purpura,  n (%) |  | 191 (100.0) | 2545 (100.0) | 188 (100.0) | 189 (100.0) |
|  | No | 191 (100.0) | 2545 (100.0) | 188 (100.0) | 189 (100.0) |
|  | Yes | 0 (0.0) | 0 (0.0) | 0 (0.0) | 0 (0.0) |
| Thrombosis,  n (%) |  | 191 (100.0) | 2545 (100.0) | 188 (100.0) | 189 (100.0) |
|  | No | 190 (99.5) | 2543 (99.9) | 188 (100.0) | 188 (99.5) |
|  | Yes | 1 (0.5) | 2 (0.1) | 0 (0.0) | 1 (0.5) |
| Arterial Thrombosis, n (%) |  | 191 (100.0) | 2545 (100.0) | 188 (100.0) | 189 (100.0) |
|  | No | 191 (100.0) | 2545 (100.0) | 188 (100.0) | 189 (100.0) |
|  | Yes | 0 (0.0) | 0 (0.0) | 0 (0.0) | 0 (0.0) |
| Venous Thrombosis, n (%) |  | 191 (100.0) | 2545 (100.0) | 188 (100.0) | 189 (100.0) |
|  | No | 191 (100.0) | 2543 (99.9) | 188 (100.0) | 188 (99.5) |
|  | Yes | 0 (0.0) | 2 (0.1) | 0 (0.0) | 1 (0.5) |
| Deep Vein Thrombosis,  n (%) |  | 191 (100.0) | 2545 (100.0) | 188 (100.0) | 189 (100.0) |
|  | No | 191 (100.0) | 2544 (100.0) | 188 (100.0) | 188 (99.5) |
|  | Yes | 0 (0.0) | 1 (0.0) | 0 (0.0) | 1 (0.5) |
| Thromboembolism,  n (%) |  | 191 (100.0) | 2545 (100.0) | 188 (100.0) | 189 (100.0) |
|  | No | 190 (99.5) | 2545 (100.0) | 188 (100.0) | 189 (100.0) |
|  | Yes | 1 (0.5) | 0 (0.0) | 0 (0.0) | 0 (0.0) |
| Pulmonary Embolism, n (%) |  | 191 (100.0) | 2545 (100.0) | 188 (100.0) | 189 (100.0) |
|  | No | 190 (99.5) | 3112 (100.0) | 188 (100.0) | 189 (100.0) |
|  | Yes | 1 (0.5) | 0.0 (0.0) | 0.0 (0.0) | 0.0 (0.0) |
| Myocardial Infarction, n (%) |  | 191 (100.0) | 2545 (100.0) | 188 (100.0) | 189 (100.0) |
|  | No | 190 (99.5) | 2545 (100.0) | 188 (100.0) | 189 (100.0) |
|  | Yes | 1 (0.5) | 0 (0.0) | 0 (0.0) | 0 (0.0) |
| Stroke, n (%) |  | 191 (100.0) | 2545 (100.0) | 188 (100.0) | 189 (100.0) |
|  | No | 190 (99.5) | 2545 (100.0) | 188 (100.0) | 189 (100.0) |
|  | Yes | 1 (0.5) | 0 (0.0) | 0 (0.0) | 0 (0.0) |
| Bleeding, n (%) |  | 191 (100.0) | 2545 (100.0) | 188 (100.0) | 189 (100.0) |
|  | No | 186 (97.4) | 2518 (98.9) | 183 (97.3) | 188 (99.5) |
|  | Yes | 5 (2.6) | 27 (1.1) | 5 (2.7) | 1 (0.5) |
| Postpartum Hemorrhage, n (%) |  | 191 (100.0) | 2545 (100.0) | 188 (100.0) | 189 (100.0) |
|  | No | 188 (98.4) | 2482 (97.5) | 182 (96.8) | 186 (98.4) |
|  | Yes | 3 (1.6) | 63 (2.5) | 6 (3.2) | 3 (1.6) |
| WHO Score, n (%) |  | 191 (100.0) | 2545 (100.0) | 188 (100.0) | 189 (100.0) |
|  | 2 | 0 (0.0) | 1 (0.0) | 0 (0.0) | 0 (0.0) |
|  | 3 | 128 (67.0) | 2459 (96.6) | 181 (96.3) | 178 (94.2) |
|  | 4 | 50 (26.2) | 81 (3.2) | 5 (2.7) | 9 (4.8) |
|  | 5 | 7 (3.7) | 4 (0.2) | 2 (1.1) | 2 (1.1) |
|  | 6 | 1 (0.5) | 0 (0.0) | 0 (0.0) | 0 (0.0) |
|  | 7 | 2 (1.0) | 0 (0.0) | 0 (0.0) | 0 (0.0) |
|  | 8 | 3 (1.6) | 0 (0.0) | 0 (0.0) | 0 (0.0) |
| Oxygen Assistance, n (%) |  | 191 (100.0) | 2545 (100.0) | 188 (100.0) | 189 (100.0) |
|  | High-Flow | 7 (3.7) | 4 (0.2) | 1 (0.5) | 1 (0.5) |
|  | Low-Flow | 50 (26.2) | 87 (3.4) | 8 (4.3) | 10 (5.3) |
|  | None | 134 (70.2) | 2454 (96.4) | 179 (95.2) | 178 (94.2) |
| Vasopressor Use, n (%) |  | 191 (100.0) | 2545 (100.0) | 188 (100.0) | 189 (100.0) |
|  | No | 184 (96.3) | 2484 (97.6) | 184 (97.9) | 182 (96.3) |
|  | Yes | 7 (3.7) | 61 (2.4) | 4 (2.1) | 7 (3.7) |
| COVID-19 Severity, n (%) |  | 191 (100.0) | 2545 (100.0) | 188 (100.0) | 189 (100.0) |
|  | Mild | 123 (64.4) | 2377 (93.4) | 166 (88.3) | 157 (83.1) |
|  | Moderate | 55 (28.8) | 164 (6.4) | 21 (11.2) | 31 (16.4) |
|  | Severe | 10 (5.2) | 4 (0.2) | 1 (0.5) | 1 (0.5) |
|  | Deceased | 3 (1.6) | 0 (0.0) | 0 (0.0) | 0 (0.0) |
| Maternal Death, n (%) |  | 191 (100.0) | 2545 (100.0) | 188 (100.0) | 189 (100.0) |
|  | No | 188 (98.4) | 2545 (100.0) | 188 (100.0) | 189 (100.0) |
|  | Yes | 3 (1.6) | 0 (0.0) | 0 (0.0) | 0 (0.0) |
| Days in Hospital After COVID-19 diagnosis (days), mean (SD) |  | 9.5 (7.8) | 5.3 (7.9) | 5.5 (6.4) | 6.6 (10.4) |
| Diagnosis Count After COVID-19 diagnosis, mean (SD) |  | 9.3 (10.1) | 2.2 (4.9) | 4.3 (8.0) | 4.6 (7.6) |
| Unique Medication Ingredient Count After COVID-19 diagnosis, mean (SD) |  | 53.3 (13.4) | 40.7 (10.6) | 43.4 (16.1) | 46.3 (17.5) |
| Gestational Age (days), mean (SD) |  | 262.3 (25.3) | 271.2 (15.5) | 263.2 (30.2) | 263.1 (27.4) |
| Preterm Birth, n (%) |  | 191 (100.0) | 2545 (100.0) | 188 (100.0) | 189 (100.0) |
|  | No | 144 (75.4) | 2250 (88.4) | 150 (79.8) | 148 (78.3) |
|  | Yes | 47 (24.6) | 295 (11.6) | 38 (20.2) | 41 (21.7) |
| Infant Birth Weight (oz), mean (SD) |  | 109.1 (29.8) | 115.1 (21.0) | 109.8 (33.3) | 108.9 (31.2) |
| Low Birth Weight, n (%) |  | 190 (100.0) | 2539 (100.0) | 188 (100.0) | 187 (100.0) |
|  | No | 154 (81.1) | 2306 (90.8) | 158 (84.0) | 157 (84.0) |
|  | Yes | 36 (18.9) | 233 (9.2) | 30 (16.0) | 30 (16.0) |
| Fetal Growth Percentile, mean (SD) |  | 50.2 (30.6) | 46.0 (28.9) | 48.4 (29.9) | 45.9 (29.9) |
| Small for Gestational Age, n (%) |  | 190 (100.0) | 2539 (100.0) | 188 (100.0) | 187 (100.0) |
|  | No | 163 (85.3) | 2174 (85.4) | 161 (85.6) | 158 (83.6) |
|  | Yes | 28 (14.7) | 371 (14.6) | 27 (14.4) | 31 (16.4) |
| Stillbirth, n (%) |  | 191 (100.0) | 2545 (100.0) | 188 (100.0) | 189 (100.0) |
|  | No | 189 (99.0) | 2526 (99.3) | 180 (95.7) | 182 (96.3) |
|  | Yes | 2 (1.0) | 19 (0.7) | 8 (4.3) | 7 (3.7) |
| Delivery Method, n (%) |  | 191 (100.0) | 2545 (100.0) | 188 (100.0) | 189 (100.0) |
|  | C-section | 101 (52.9) | 885 (34.8) | 84 (44.7) | 76 (40.2) |
|  | Vaginal | 90 (47.1) | 1660 (65.2) | 104 (55.3) | 113 (59.8) |

The observation endpoint was the delivery date, except for postpartum hemorrhage. Variables are defined in Table S1. SNOMED codes of diagnoses are presented in Table S2.

## Table S9 Outcome comparison between treatment group and control, matched control, and sensitivity analysis matched control group

|  | **Treatment group** | | **Control group** | | | **Matched control group** | | | | **Sensitivity analysis matched control group** | | | |
| --- | --- | --- | --- | --- | --- | --- | --- | --- | --- | --- | --- | --- | --- |
| **Outcome** | **Case (prevalence rate, 95% CI)** | **Case (prevalence rate, 95% CI)** | | **Relative risk, 95% CI** | ***P*-value** | | **Case (prevalence rate, 95% CI)** | **Relative risk, 95% CI** | ***P*-value** | | **Case (prevalence rate, 95% CI)** | **Relative risk, 95% CI** | ***P*-value** |
| Coagulopathy | 2 (0.011, [0.003,0.037] | 9 (0.004, [0.002,0.007] | | 3.0 [0.6,13.6] | 0.18 | | 2 (0.011, [0.002,0.038] | 1.0 [0.1,6.9] | 1.0 | | 1 (0.005, [0.001,0.029] | 2.0[0.2,21.6] | 1.0 |
| Bleeding | 5 (0.026, [0.011,0.06] | 27 (0.011, [0.007,0.015] | | 2.5 [1.0,6.3] | 0.07 | | 5 (0.027, [0.007,0.061] | 1.0 [0.3,3.3] | 1.0 | | 1 (0.005, [0.001,0.029] | 5.0 [0.6,42.0] | 0.22 |
| Postpartum hemorrhage | 3 (0.016, [0.005,0.045] | 63 (0.025, [0.019,0.032] | | 0.6 [0.2,2.0] | 0.62 | | 6 (0.032, [0.019,0.068] | 0.5 [0.1,1.9] | 0.34 | | 3 (0.016, [0.005,0.046] | 1.0 [0.2,4.8] | 1.0 |
| O2 assistance | 63 (0.33, [0.273,0.387] | 91 (0.036, [0.029,0.044] | | 9.2 [6.9,12.3] | <.001 | | 9 (0.048, [0.029,0.088] | 6.9 [3.5,13.5] | <.001 | | 11 (0.058, [0.033,0.101] | 5.7 [3.1,10.4] | <.001 |
| Vasopressor use | 7 (0.037, [0.018,0.074] | 61 (0.024, [0.019,0.031] | | 1.5 [0.7,3.3] | 0.33 | | 4 (0.021, [0.019,0.053] | 1.7 [0.5,5.8] | 0.54 | | 7 (0.037, [0.018,0.074] | 1.0 [0.4,2.8] | 1.0 |
| Death | 3 (0.016, [0.005,0.045] | 0 (0.0, [0.0,0.002] | | NA | <.001 | | 0 (0.0, [0.0,0.02] | NA | 0.25 | | 0 (0.0, [0.0,0.02] | NA | 0.25 |
| Stillbirth | 2 (0.011, [0.003,0.037] | 19 (0.007, [0.005,0.012] | | 1.4 [0.3,6.0] | 0.65 | | 8 (0.043, [0.005,0.082] | 0.2 [0.0,1.1] | 0.06 | | 7 (0.037, [0.018,0.074] | 0.3 [0.0,1.3] | 0.10 |
| Low birth weight | 36 (0.19, [0.141,0.248] | 233 (0.092, [0.081,0.104] | | 2.1 [1.5,2.8] | <.001 | | 30 (0.16, [0.081,0.217] | 12 [0.8,1.8] | 0.50 | | 30 (0.16, [0.115,0.218] | 1.2 [0.8,1.8] | 0.50 |
| Preterm birth | 47 (0.246, [0.192,0.307] | 295 (0.116, [0.104,0.129] | | 2.1 [1.6,2.8] | <.001 | | 38 (0.202, [0.104,0.262] | 1.2 [0.8,1.8] | 0.33 | | 41 (0.217, [0.165,0.277] | 1.1 [0.8,1.6] | 0.54 |
| Small for gestational age | 28 (0.147, [0.104,0.202] | 371 (0.146, [0.133,0.16] | | 1.0 [0.7,1.4] | 1.0 | | 27 (0.144, [0.133,0.2] | 1.0 [0.6,1.7] | 1.0 | | 31 (0.164, [0.118,0.222] | 0.9 [0.6,1.4] | 0.67 |

NA: Not applicable, too few cases to calculate

|  | **Treatment group** | **Control group** | | **Matched**  **control group** | | | **Sensitivity analysis matched control group** | |
| --- | --- | --- | --- | --- | --- | --- | --- | --- |
| **Outcome** | **0, 25, 50, 75, 100 percentile** | **0, 25, 50, 75, 100 percentile** | ***P*-value** | **0, 25, 50, 75, 100 percentile** | ***P*-value** | **0, 25, 50, 75, 100 percentile** | | ***P*-value** |
| Days in hospital after COVID-19 diagnosis (days), mean (SD) | 0,4,6,11,51 | 0,0,3,4,189 | <.001 | 0,0,3,4,55 | <.0001 | 0,1,3,5,62 | | <.001 |
| Diagnosis count | 0,1,7,14,60 | 0,0,0,2,59 | <.001 | 0,0,0,5,38 | <.0001 | 0,0,1,6,33 | | <.001 |
| Medication count | 30,44,51,60,133 | 18,34,39,46,129 | <.001 | 20,34,39,50,115 | <.0001 | 20,36,44,51,115 | | <.001 |

Categorical and continuous outcome variables were evaluated using Fisher’s exact test and Mann Whitney U test.

## Table S10 Influence of maternal age and pregravid BMI on relations between prophylactic anticoagulant administration and risks of outcomes

|  | **Before adjustment** | | **After adjustment** | |
| --- | --- | --- | --- | --- |
| **Outcome** | **RR, 95% CI** | ***P*-value** | **RR, 95% CI** | ***P*-value** |
| Coagulopathy | 1.0[0.1,13.7] | 1.0 | 0.9[0.1,6.8] | 0.95 |
| Bleeding | 1.0[0.2,4.4] | 1.0 | 0.9[0.3,3.3] | 0.90 |
| Postpartum hemorrhage | 0.5[0.1,2.3] | 0.34 | 0.5[0.1,1.9] | 0.27 |
| O2 assistance | 9.7[4.6,23.1] | <.001 | 9.5[4.5,19.8] | <.001 |
| Vasopressor use | 1.7[0.4,8.3] | 0.54 | 1.8[0.5,6.4] | 0.36 |
| Death | NA[0.4,inf] | 0.25 | NA[0.0,inf] | 1.0 |
| Preterm birth | 1.3[0.8,2.2] | 0.33 | 1.3[0.8,2.1] | 0.29 |
| Small for gestational age | 1.0[0.6,1.9] | 1.0 | 1.1[0.6,1.9] | 0.82 |
| Low birth weight | 1.2[0.7,2.2] | 0.50 | 1.2[0.7,2.1] | 0.42 |
| Stillbirth | 0.2[0.0,1.2] | 0.06 | 0.3[0.1,1.5] | 0.15 |

NA: Not applicable, too few cases to calculate

# Supplementary Figures


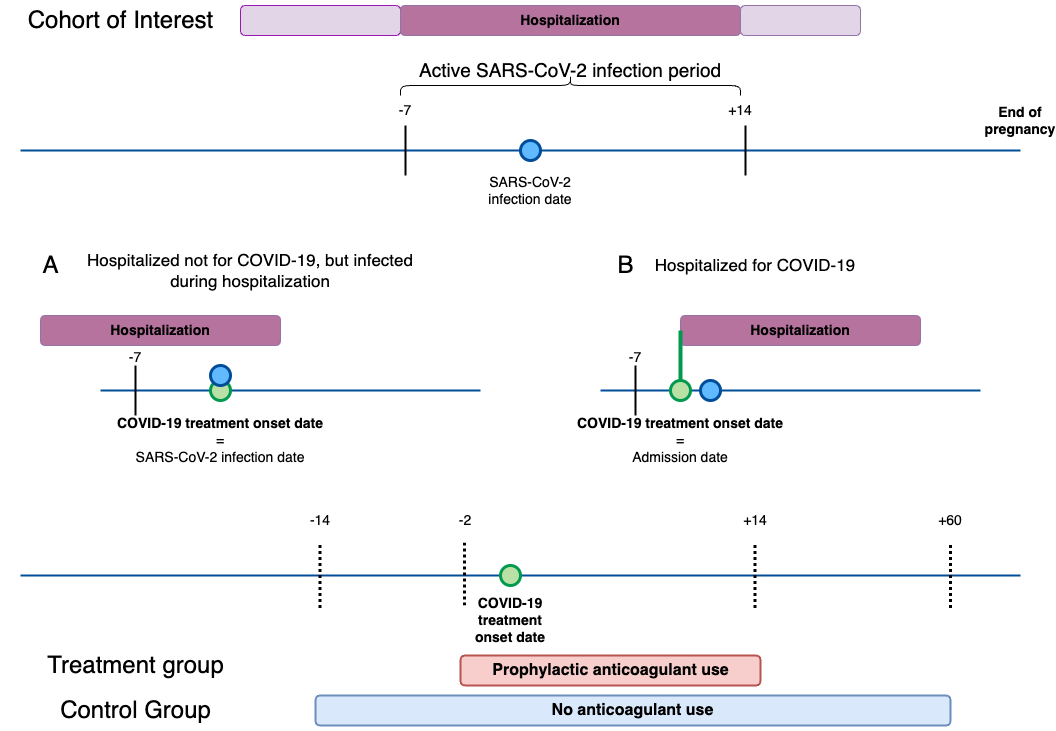


## Figure S1 Timeline and exposure group definition

Cohort of interest was defined as pregnant patients hospitalized anytime during the active SARS-CoV-2 infection. We defined the active COVID-19 infection period as -7~+14 days from the COVID-19 infection date. This date range was determined based on the COVID-19 incubation period and symptomatic period.[[30,35]](https://paperpile.com/c/KBCGQG/dwv2i+vcTRB) SARS-CoV-2 infection date was either the date of COVID-19 diagnosis or the date of the SARS-CoV-2 NAAT positive test result, whichever preceded. We considered two possible scenarios for hospitalization: A) Hospitalized not for COVID-19, but infected during hospitalization, and B) Hospitalized for COVID-19. If patients were admitted before the start of active SARS-CoV-2 infection period, we categorized them into A scenario. If not, they were categorized into B scenario. For category A, SARS-CoV-2 infection date was considered as the COVID-19 treatment onset. For category B, admission date was considered as the COVID-19 treatment onset. We used COVID-19 treatment onset as our index date for exposure, instead of the infection date, because our exposure of interest was inpatient prophylactic anticoagulant administration relevant to COVID-19 treatment. The treatment group was defined as patients who had only prophylactic anticoagulant administration during -2~+14 days from the COVID-19 treatment onset date. The control group was defined as patients who had no anticoagulant administration during -14~+60 days from the COVID-19 treatment onset date.


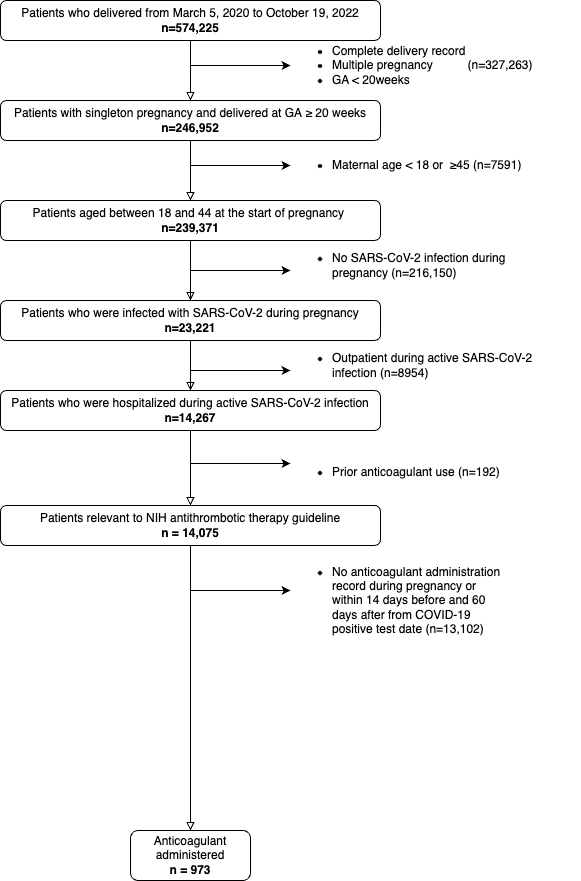


## Figure S2 Truveta cohort selection flow chart

Variables are defined in Table S1.


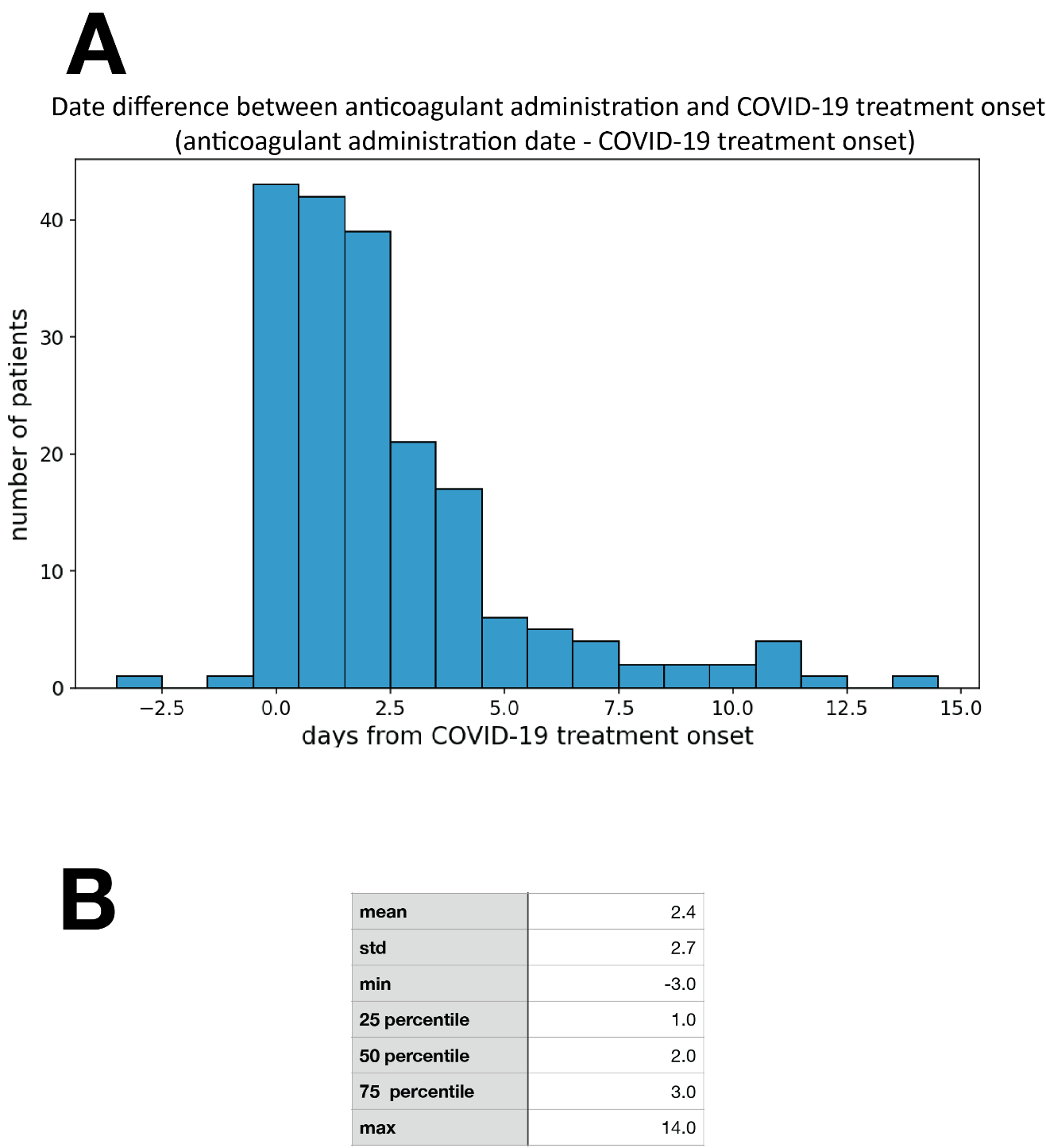


## Figure S3 Distribution of the time gap (in days) between COVID-19 treatment onset and anticoagulant administration

1. Histogram of the distribution of date difference between the prophylactic anticoagulant administration date and COVID-19 treatment onset (anticoagulant administration date - COVID-19 treatment onset)
2. Descriptive statistics (mean, standard deviation, minimum, 25 percentile, median, 75 percentile, maximum) of the distribution of the time gap between anticoagulant administration date and COVID-19 treatment onset. 75 percent of patients took anticoagulants within the first three days of the COVID-19 treatment onset date


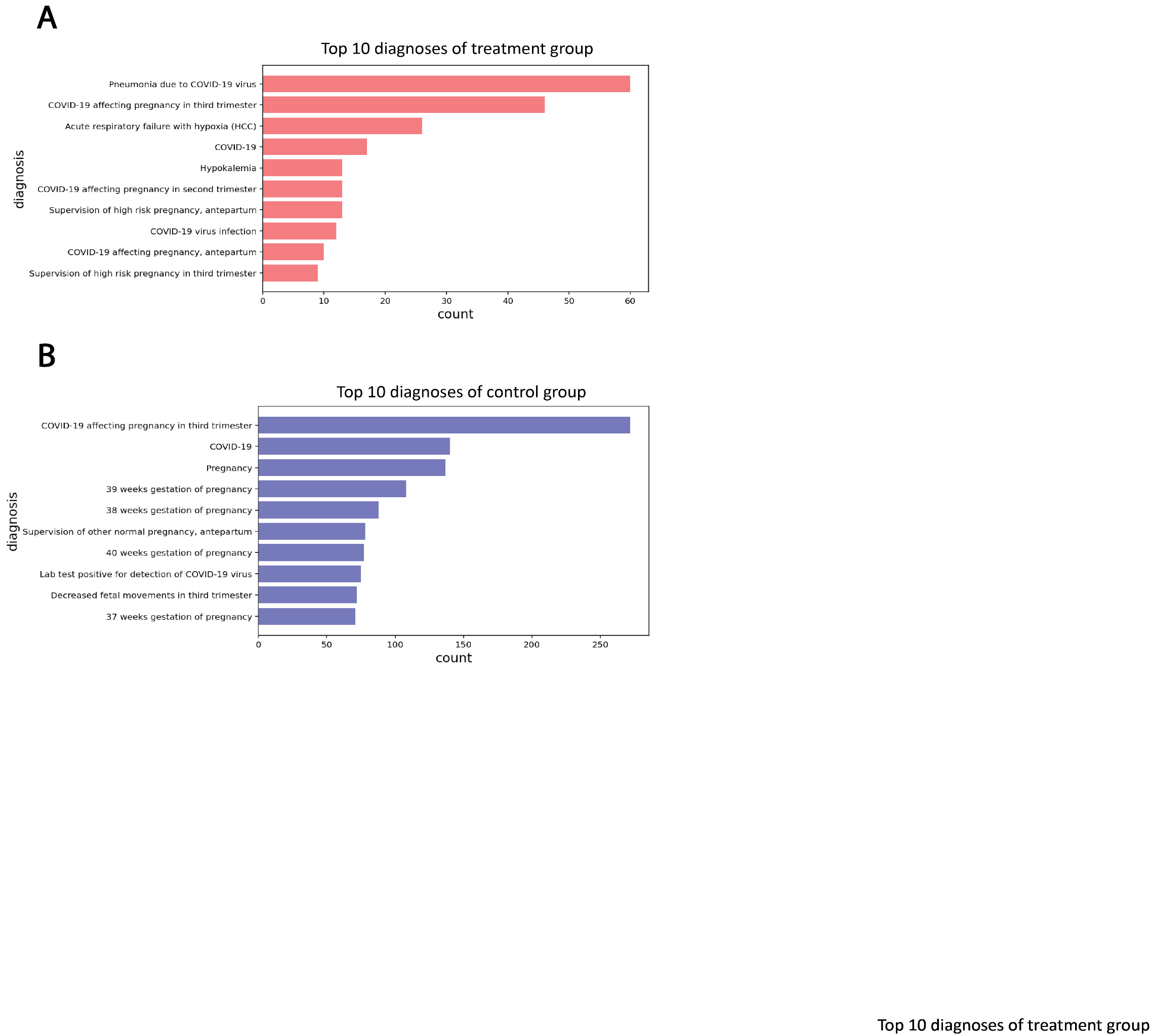


## Figure S4 Top 10 diagnoses of treatment and control group during the period between SARS-CoV-2 infection and delivery date

1. Top 10 diagnoses of the treatment group during SARS-CoV-2 infection and date of delivery. If the same diagnosis was given more than once to the patient during the study period, we counted it as one.
2. Top 10 diagnoses of the control group during SARS-CoV-2 infection and date of delivery. If the same diagnosis was given more than once to the patient during the study period, we counted it as one.


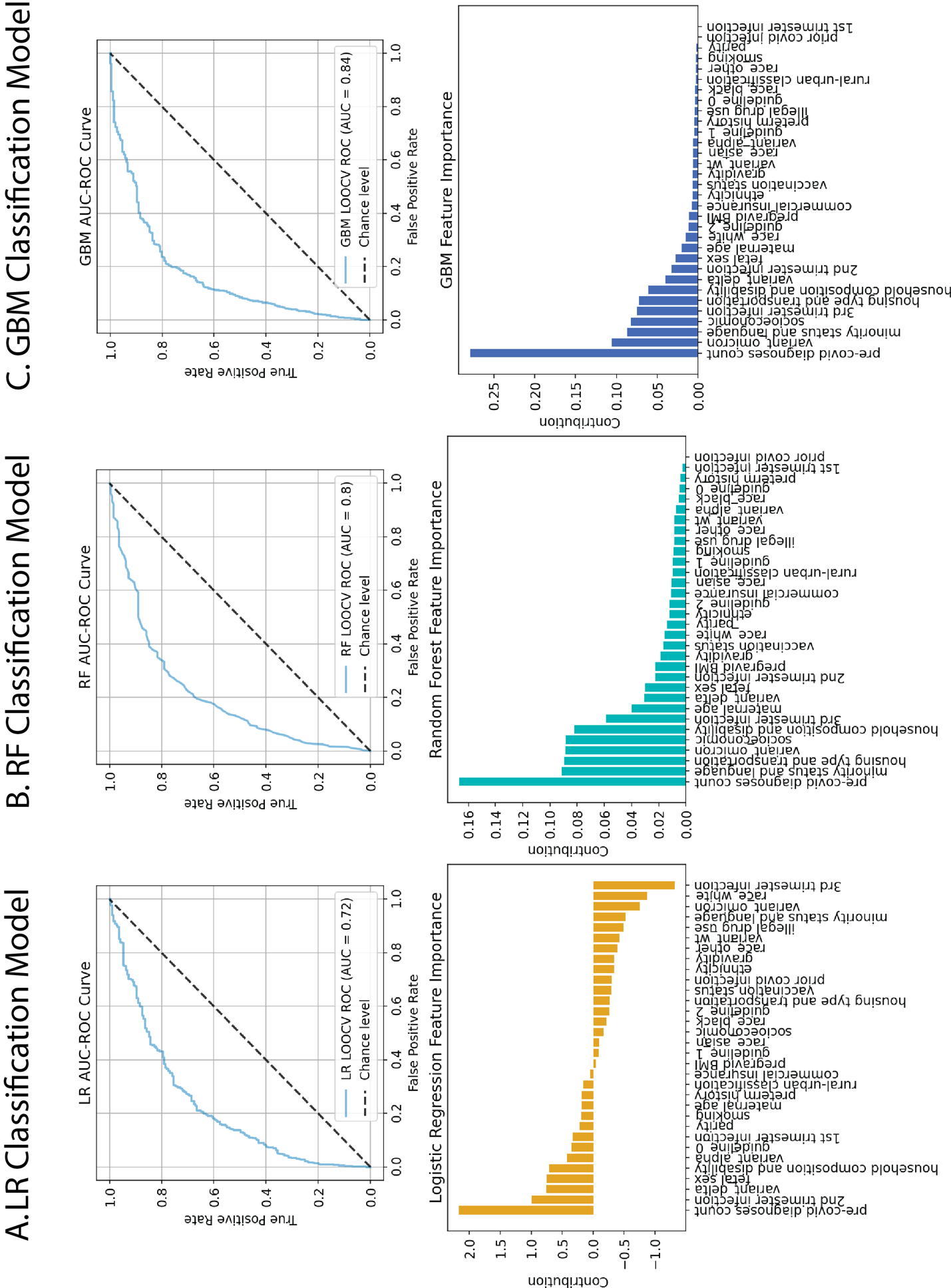


## Figure S5 Classification model performance

AUC-ROC Area under the receiver operator characteristics curve

Each model was trained with 28 features in the feature importance plot to classify prophylactic anticoagulant administration status among our cohort of interest. Impurity-based feature importance evaluated the contribution of 28 features on demographic, comorbidity, geographical, pregnancy, maternal, clinical recommendation, and SARS-CoV-2 variant characteristics. The confidence interval of AUC-ROC was calculated using DeLong’s method[[36]](https://paperpile.com/c/KBCGQG/QTTxd)

1. Logistic regression classification model performance based on AUC-ROC and feature importance plot. 95% confidence interval of AUC-ROC was [0.68, 0.75]
2. Random forest classification model performance based on AUC-ROC and feature importance plot. 95% confidence interval of AUC-ROC was [0.76, 0.83]
3. Gradient boosting machine classification model performance based on AUC-ROC and feature importance plot. 95% confidence interval of AUC-ROC was [0.81, 0.87]


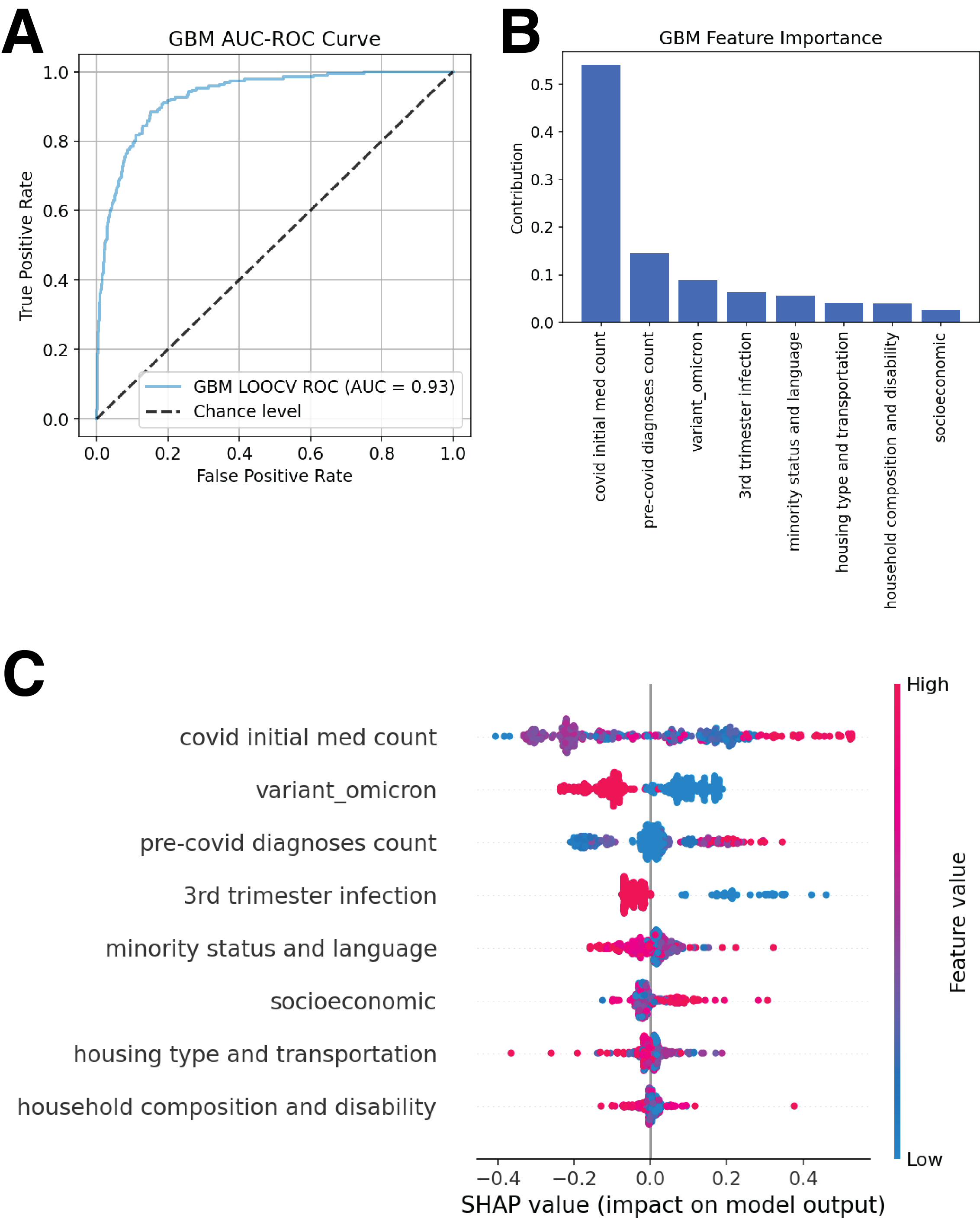


## Figure S6 Sensitivity analysis result of classification model performance, feature importance, and propensity score matching result

We performed a sensitivity analysis to assess the impact of COVID-19 illness severity at the time of COVID-19 treatment onset. The definition of COVID-19 initial medication count is the count of unique medication ingredients -3-+3 days from the COVID-19 treatment onset. We used this variable to reflect the COVID-19 illness severity. This date range was determined based on the distribution of the time gap between COVID-19 treatment onset and anticoagulant administration date. 75 percentile patients were exposed to anticoagulants within the first three days from the COVID-19 treatment onset date. We used the model with the best performance from the main analysis, the gradient boosting machine classification model. We trained this model with the seven most important features from the main analysis and results were as follows.

1. Gradient boosting machine classification model performance based on AUC-ROC. 95% confidence interval of AUC-ROC was [0.91, 0.95].
2. Feature importance ranking of the limited model. Variables are defined in Table S1. COVID-19 initial medication count contributed the most toward classifying prophylactic anticoagulant administration status.
3. Shapley permutation explainer of the feature contribution (Supplemental Method). SHAP value reflects the contribution of the seven most important features from the gradient boosting models towards classifying prophylactic anticoagulant administration status. SHAP value is the average marginal contribution of a feature value across all permutations of features. Each row represents an individual feature, and the dot represents a sample. The dot color reflects the value of the feature of the sample relative to all samples. The evaluation was done on the sample set comprised of a treatment group and a 1:1 randomly undersampled matched control group (n=382). For the row of COVID-19 initial medication count, red and blue dots were spread out across extreme negative SHAP values and up to 0.2 SHAP value, but the end of the positive SHAP axis (SHAP value > 0.2) was clustered with red dots. This describes a positive correlation between COVID-19 initial medication count and prophylactic anticoagulant administration status.

**
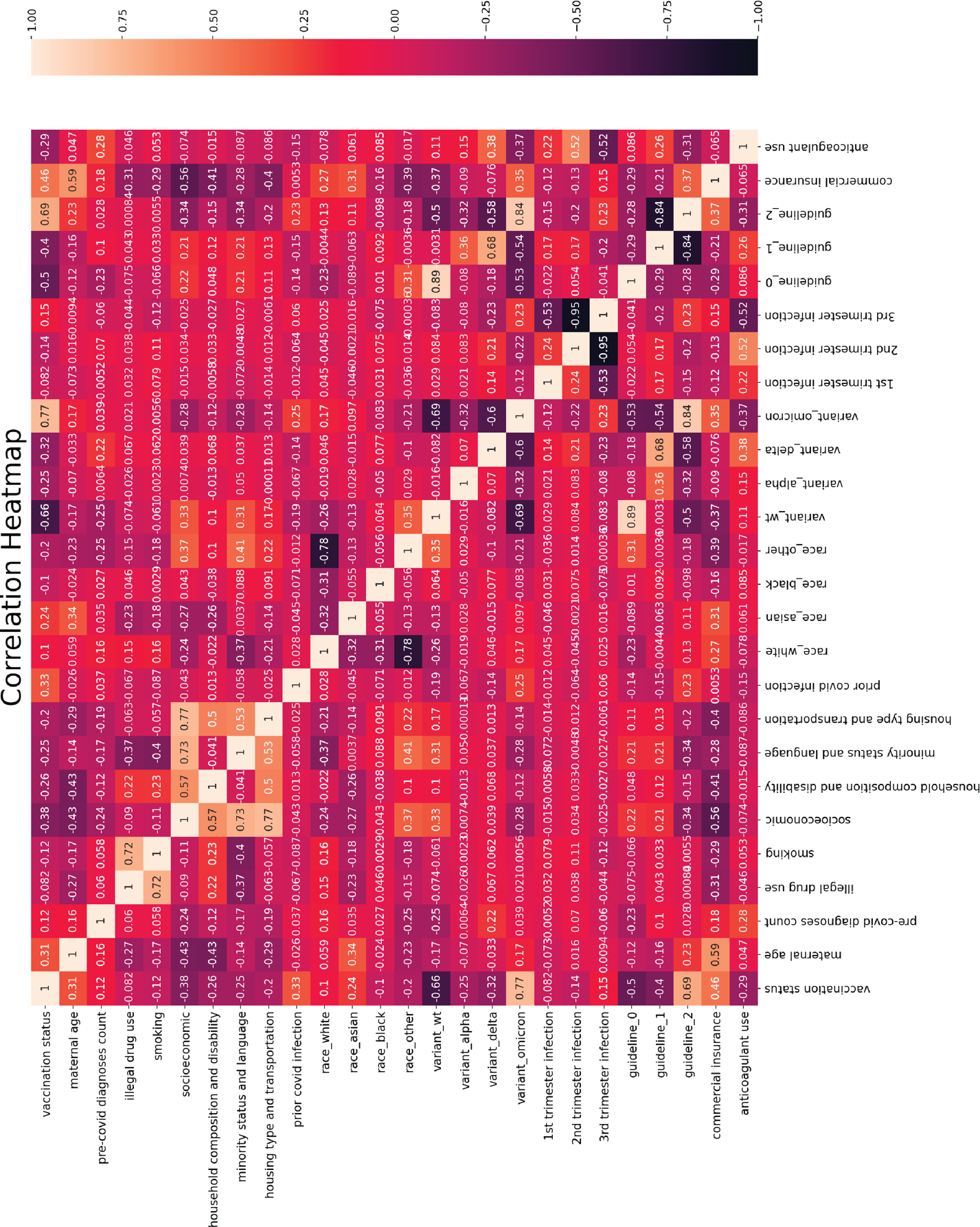
**

## Figure S7 Pairwise Pearson correlation plot between variables

Variables are defined in Table S1. The Pearson correlation has a value of [-1,1] and measures the strength of the linear relationship between variables. A negative value means a negative linear correlation, and a positive value means a positive one. 0 means no linear correlation


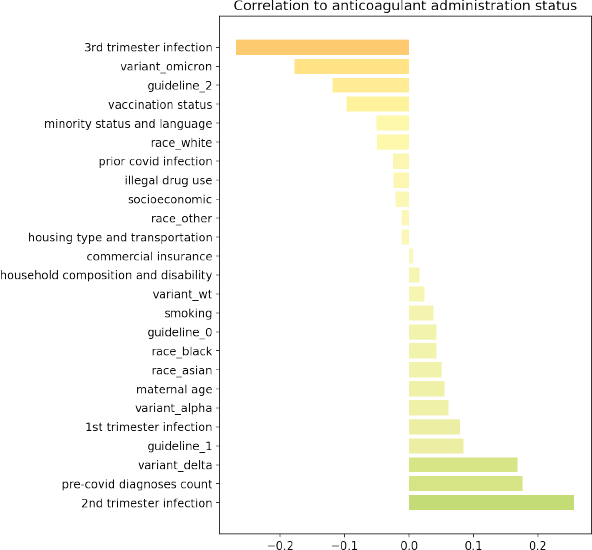


## Figure S8. Pearson correlation between individual variables and prophylactic anticoagulant administration status

Variables are defined in Table S1. The Pearson correlation has a value of [-1,1] and measures the strength of the linear relationship between variables. A negative value means a negative linear correlation, and a positive value means a positive one. 0 means no linear correlation.
